# Primary care and community interventions for multimorbidity involving depression or anxiety: systematic review with meta-analysis

**DOI:** 10.1136/bmjmed-2025-002400

**Published:** 2026-04-10

**Authors:** Kieran Sweeney, Michaela Gilarova, Lauren Ng, Jennifer Baker, Susanne Maxwell, Clare Macrae, Stewart W Mercer, Atul Anand, Bruce Guthrie, Lucy E Stirland

**Affiliations:** 1 University of Edinburgh Usher Institute, Edinburgh, UK; 2 Advanced Care Research Centre, University of Edinburgh Usher Institute, Edinburgh, UK; 3 NHS Education for Scotland South East Region, Edinburgh, UK; 4 University of Edinburgh Centre for Cardiovascular Science, Edinburgh, UK; 5 Institute for Neuroscience and Cardiovascular Research, University of Edinburgh Division of Psychiatry, Edinburgh, UK; 6 Global Brain Health Institute, San Francisco, California, USA

**Keywords:** Mental health, Primary health care

## Abstract

**Objective:**

To identify and characterise primary care or community based interventions for patients with multimorbidity involving depression or anxiety, and to determine their effectiveness for improving patients’ mental health, physical health, and quality of life.

**Design:**

Systematic review with meta-analysis.

**Data sources:**

Medline, Embase, Cochrane Library, CINAHL, PsycInfo, and Web of Science databases, from inception to 11 November 2024.

**Eligibility criteria for selecting studies:**

Included studies were randomised controlled trials of primary care or community based interventions targeting adults with depression or anxiety disorders and one or more long term physical conditions. Risk of bias assessment used the Cochrane risk of bias tool. Interventions were categorised as organisational or patient oriented, and were subgrouped by intervention type. Intervention components were systematically categorised, and effects on mental health and quality of life outcomes were meta-analysed in groups defined by intervention type and assessment time point. Physical health outcomes were too heterogenous to meta-analyse and were synthesised without meta-analysis with Fisher's method for combining P values.

**Results:**

29 randomised controlled trials comprising 9487 participants were included. High quality evidence was found for organisational interventions (n=10, including collaborative care, stepped care, and post-discharge interventions) which resulted in small improvements in symptoms of depression (standardised mean difference −0.25, 95% confidence interval (CI) −0.43 to −0.06) and quality of life (0.21, 0.01 to 0.41), but had no effect on symptoms of anxiety at the end of the intervention. No effect on depression or anxiety symptoms was observed, and no data for quality of life were found from organisational interventions at the late follow-up period (18-24 months). In the subgroup analysis, collaborative care resulted in sustained improvements in symptoms of depression at 18-24 months. Synthesis without meta-analysis showed evidence of benefit from organisational interventions (specifically collaborative care) on physiological (eg, haemoglobin A_1c_ levels), but not on functional (eg, disability) or global physical health outcomes. Low to moderate quality evidence was found for patient oriented interventions (n=19; interventions including exercise, psychotherapy, and psychoeducation) which led to small improvements in symptoms of depression (standardised mean difference −0.46, 95% CI −0.71 to −0.21) and quality of life (0.22, 0.14 to 0.29) at the end of the intervention. These effects were diminished at the late follow-up period (≥12 months). In the subgroup analysis, no reported data for the long term effects of exercise, psychotherapy, or psychoeducation (18-24 months after randomisation) were found. Synthesis without meta-analysis showed evidence of benefit from patient oriented interventions (primarily psychotherapy) on physiological, functional, and global physical health outcomes.

**Conclusions:**

The study showed that interventions improved mental health, physical health, and quality of life outcomes in people with multimorbidity involving depression or anxiety, but the effects were small and, for patient oriented interventions in particular, diminished over time.

**Systematic review registration:**

PROSPERO CRD420251004355.

**WHAT IS ALREADY KNOWN ON THIS TOPIC**

- The coexistence of depression or anxiety with long term physical conditions is associated with poor health outcomes
- Uncertainty exists about the form and effectiveness of interventions in these patients, with most research examining specific pairs of conditions

**WHAT THIS STUDY ADDS**

- Across 29 studies, two main groups of interventions (organisational and patient oriented) were identified, and their components systematically characterised
- Both interventions resulted in small short term improvements in mental health and quality of life
- Little effects were seen at later follow-up times, with limited benefits for physical health outcomes

**HOW THIS STUDY MIGHT AFFECT RESEARCH, PRACTICE OR POLICY**

- Future interventions should focus on optimising long term improvements in mental and physical health, while avoiding the complex service redesign and resource requirements that have proven an implementation barrier for some organisational interventions

## Introduction

 Mental-physical multimorbidity, defined as multiple or coexisting long term physical and mental health conditions, has a substantial impact on people and healthcare services, particularly in more socioeconomically deprived areas where it is more common and occurs much earlier in life.^1 2^ A large portion of the burden of mental-physical multimorbidity comes from common mental disorders, such as depression and anxiety disorders, which are a leading cause of disability^3^ and a major driver of healthcare use and costs in people with multimorbidity.^4^ Globally, an estimated 6% of adults have depression,^5^ and the risk is 2-3 times higher in those with chronic physical conditions.^2^ Having both physical and mental health conditions is associated with poorer quality of life and physical health outcomes.^6^ The cost to society, through loss of social and workforce participation, is also substantial.^7^ Interventions aimed at this group are therefore a priority for patients and policy makers.^8^,^11^ Moreover, while uncertainties remain about the effectiveness of interventions for people with multimorbidity in general,^12 13^ a more targeted approach focusing on individuals with mental-physical multimorbidity could improve health outcomes and reduce inequalities.

The close association between common mental disorders and chronic physical conditions is shown by their bidirectional and cumulative relation. Evidence from multimorbidity studies has indicated a dose-response relation between number of physical conditions and the risk of depression, as well as a faster rate of accrual of physical conditions in people with depression.^14 15^ Many physical conditions also share common risk factors and causal pathways with depression.^16^ This association not only supports the argument for more integrated care, but also implies common ground for intervention. A number of interventions have shown potential for beneficial effects on physical and mental health, including strategies based on the collaborative care model, self-management principles, psychological treatments (particularly cognitive behavioural therapy), and physical activity.^17^,^21^ Despite global efforts to promote more integrated care,^16 22^ management of chronic disease remains fragmented and directed towards single diseases, while efforts to integrate care have faced numerous barriers.^23^

Previous systematic reviews in this area have focused on specific comorbidity pairs,^24^,^26^ specific interventions,^27 28^ or on a range of interventions for multimorbidity in patients who may not have depression or anxiety.^12^ This review aimed to identify a variety of interventions for people with multimorbidity involving depression or anxiety, to characterise intervention components, and to evaluate and compare their effectiveness for improving mental health, physical health, and quality of life outcomes.

## Methods

This systematic review followed Preferred Reporting Items for Systematic Reviews and Meta-Analyses (PRISMA) guidance.^29^ We conducted a systematic search of six databases from inception to 11 November 2024: Medline, Embase, Cochrane Library, CINAHL, PsycInfo, and Web of Science. We developed the search strategy iteratively with the support of an academic support librarian and with reference to previous reviews.^12 30^ The final search terms covered key concepts of multimorbidity, chronic physical conditions, depression, and anxiety disorders (online supplemental box S1). The search was restricted to English language publications. We identified additional relevant studies by reviewing studies cited by previous relevant systematic reviews and clinical guidelines,^1821 27 28 31^,^33^ and by using Scopus (February 2025) to review forward and backward citations of included studies. A review protocol was prospectively registered on PROSPERO (CRD420251004355).^34^

### Inclusion criteria

This review included studies of randomised control trials in which the intervention was targeted at adults (≥18 years) with symptoms of depression or anxiety, or both (determined by a formal diagnosis, or by symptoms above a stated threshold on a validated screening tool), and at least one chronic physical condition. No limitations were considered on the type of physical conditions that participants could have, but we required trials to list or allow more than one physical condition within their eligibility criteria to be included in the review. We chose this design to identify interventions aimed towards multimorbidity, with potential for adaptability across different physical conditions, as opposed to interventions only looking at specific comorbidity pairs (eg, depression with cancer, or anxiety with asthma).

We included interventions based in any community or primary care setting, including remote or digital interventions, home based interventions, and interventions based in outpatient services. Interventions given within day hospital or inpatient settings were excluded. Interventions could include any treatment, strategy, or model of care aimed at patients in the defined population, excluding interventions that comprised drug treatments only. To be included in the review, trials had to compare the intervention with usual care, no treatment, or an attention control. Usual care was defined as the standard clinical management available to the study population in the given setting. Use of antidepressants was considered usual care, available to the intervention and control groups, but interventions could include strategies to increase adherence, or to enhance the review and titration of drug treatments. Outcomes of interest were the three domains, mental health, quality of life, and physical health, at least one of which had to be reported for a study to be eligible for inclusion. The specific outcome measures included in these three domains are detailed below. Studies reporting only economic analyses were excluded.

### Study selection

Identified titles and abstracts, and subsequently full text papers, were independently screened by two reviewers, with disagreements at both stages resolved by consensus. KS screened all studies at both stages, and MG, LN, JB, and SM were second screeners.

### Data extraction

We used a bespoke form to extract the key characteristics of each study, such as year of publication, setting, and country, duration and type of intervention, key eligibility criteria (age, mental health conditions, and physical conditions), participant characteristics (sex, age, and socioeconomic position), sample size, and relevant outcomes. Two reviewers conducted the initial data extraction (KS with a second reviewer) to refine the extraction form (ie, number of variable columns) and ensure consistency in the level of detail and terminology. Two thirds of subsequent data extraction undertaken by KS was then checked by a second reviewer, who independently reviewed the study results to identify any discrepancies to be resolved by consensus. Where relevant data was not reported, the corresponding author was contacted.

### Intervention components

The components of each intervention were systematically categorised, informed by the taxonomy of health systems interventions published by the Cochrane Effective Practice and Organisation of Care Review Group^35^ and the SELFIE (sustainable integrated chronic care models for multi-morbidity: delivery, financing, and performance) framework for integrated care for multimorbidity,^36^ adapted to the range and type of components in the included studies.

### Meta-analysis of intervention effects: mental health and quality of life outcomes

We performed a meta-analysis for the effect of interventions on mental health (symptoms of depression and anxiety) and quality of life outcomes, reported using continuous measures. Online supplemental tables S1-S3 describe in full the outcome measures that were meta-analysed. For depression, these included the Patient Health Questionnaire 9 item scale, Symptoms Checklist 13 item and 20 item scales, and Geriatric Depression Scale.^37^,^39^ For anxiety, these included the Generalised Anxiety Disorder 7 item scale, Hospital Anxiety and Depression Scale-Anxiety Subscale, and Geriatric Anxiety Inventory.^40^,^42^ Depression and anxiety were commonly and consistently reported across studies, enabling meta-analysis of these precise constructs. In contrast, measures of distress and mental component subscales from wellbeing measures were not included because they were more variably measured and reported. For quality of life, only generic measures were extracted and synthesised, including European Quality of Life group (EuroQol) 5 Dimensions 5 level version, EuroQol Visual Analogue Scale, and Short Form Health Survey 12 item and 36 item versions.^43^,^45^ We did not include disease specific quality of life measures. Where a study reported results based on two different measures for the same outcome (eg, where both the Symptoms Checklist 13 item scale and Patient Health Questionnaire 9 item scale were reported for depression), the result for the primary outcome measure was included in meta-analysis or, if neither were the primary outcome, the measure most commonly reported in other studies.

We used the metafor package in R version 2025.05.1 to perform the meta-analysis.^46 47^ For each study and each outcome, an estimate of the effect of the intervention was calculated based on the difference between the mean outcome scores of the intervention and control groups at the relevant assessment point. We estimated this effect as a standardised mean difference (the mean difference divided by the pooled standard deviation) to account for the fact that different outcome measures for the same construct (eg, Patient Health Questionnaire 9 item scale and Geriatric Depression Scale for depression) have different scales. Where studies reported standardised mean difference with associated variance, these data were extracted directly (adjusted if reported, unadjusted if not), as recommended by Cochrane guidance.^48^ We calculated the pooled estimate of effect for the studies in each meta-analysis with a random effects model to account for heterogeneity among studies. Studies only reporting effect based on dichotomous outcomes (such as the proportion of participants achieving a specified level of symptom improvement) were not included in the meta-analysis.

Consistent with the protocol, we divided the meta-analysis into groups based on intervention type because one synthesis across heterogeneous intervention categories was not theoretically justifiable or meaningful. Interventions were first categorised as organisational or patient oriented, in line with previous systematic reviews of multimorbidity interventions and informed by the Cochrane Effective Practice and Organisation of Care taxonomy.^12 35^ We determined the categorisation of each intervention by discussion among the review team, based on study characteristics, before synthesis. For the meta-analysis, studies were also grouped by follow-up assessment time point, defined for the primary analysis as either end intervention (the closest assessment time point to the end of the active intervention) or late follow-up. We determined these time frames separately for each intervention group according to commonly reported assessment time points. For organisational interventions, end intervention was 4-12 months after randomisation and late follow-up was 18-24 months. For patient oriented interventions, end intervention was three weeks to four months after randomisation, and late follow-up was 12 months. We excluded studies at high risk of bias from the per protocol sensitivity analysis.

### Subgroup analysis

In the organisational and patient oriented intervention groups, we defined subgroups by the central model or component of the intervention (online supplemental box S2). For patient oriented interventions, subtypes were exercise, psychoeducation, and psychotherapy. For organisational interventions, subtypes were collaborative care, post-discharge, and stepped care interventions. We used two consistent time frames across all intervention subtypes to directly compare interventions, regardless of duration: short term (3-12 months after randomisation) and long term (18-24 months after randomisation). If more than one result was reported for a single study within a given time frame, the latest result was used. Beyond subgroup analysis, exploring heterogeneity further with meta-regression was not possible because of insufficient data.

### Synthesis without meta-analysis: physical health outcomes

Meta-analysis was not possible for the effect of interventions on physical health outcomes because of the diversity of outcomes in this category and the inconsistency of reported information (beyond P values and direction of effect). Assessing whether interventions improved physical health outcomes was nevertheless an important objective of this study, as agreed with the patient and public involvement group. We therefore synthesised effects on physical health outcomes by using Fisher's method for combining P values, with the metap package in R,^49^ and visualised the effects with an Albatross plot.^50^ Physical health outcomes at end intervention were grouped into three categories: global (eg, physical wellbeing and self-reported physical health), functional (eg, WHO-Disability Assessment Schedule^51^ and Sheehan's Disability Scale^52^), and physiological (eg, haemoglobin A_1c_ levels, blood pressure, and lipid levels). For mental health and quality of life outcomes, when more than one outcome was reported within a category (eg, multiple physiological outcomes), we chose the primary outcome for the trial or the outcome most commonly reported in other studies in the review for synthesis. Analysis was grouped by intervention type.

### Assessment of risk of bias

We used the Cochrane risk of bias (RoB-V2) tool to assess risk of bias for randomised controlled trials (or cluster randomised controlled trials where appropriate), summarised graphically with the ROBVIS (risk of bias visualisation) tool.^53 54^ Assessment covered the following domains: bias arising from the randomisation process, bias caused by deviations from the intended interventions, bias caused by missing outcome data, bias in measurement of the outcome, and bias in selection of the reported result. According to the RoB-V2 tool, the suggested overall risk of bias is determined by the worst score in any of the five domains, although overall rating can be upgraded or downgraded if deemed appropriate. Studies were classified as having a high risk of bias overall if any of these five domains assessed were considered high risk. KS assessed all of the studies, two thirds of the studies were independently assessed by a second reviewer (MG or CM), and any disagreements were resolved by consensus. We examined funnel plots if ≥10 studies were available, with Egger's test used to evaluate small study bias.

### Assessment of evidence quality

We assessed the overall quality (or certainty) of the evidence for each meta-analysis with the Grading of Recommendations Assessment, Development, and Evaluation approach.^55^ Assessment included consideration of risk of bias, inconsistency, indirectness, imprecision, and publication bias. An initial rating of high quality evidence was downgraded by one level for serious concerns (or by two levels for very serious concerns) in each of these domains.

### Patient and public involvement

This review benefited from the input of a patient and public involvement group established to support this and related work, made up of five people with mental-physical multimorbidity. Meeting before, during, and after the review process, group members considered the value of the research question being investigated, helped prioritise the outcomes being examined, and helped make sense of the findings from a lived experience perspective.

## Results

**Figure 1:**
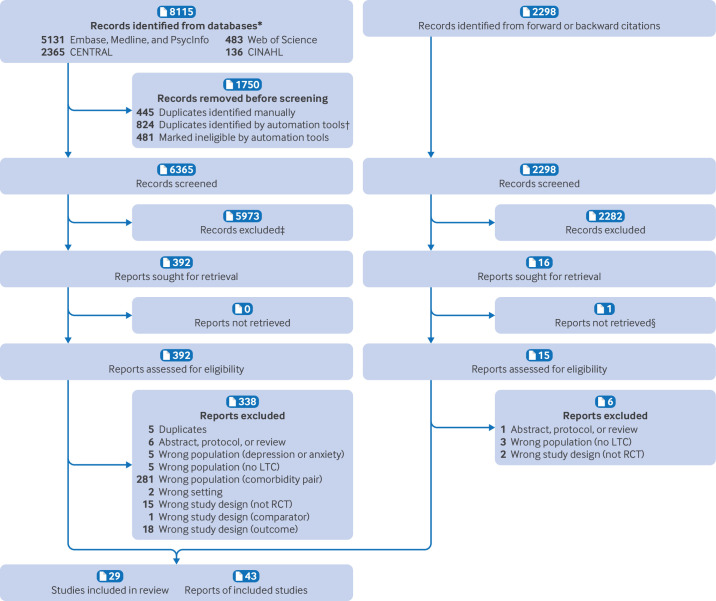
Preferred Reporting Items for Systematic Reviews and Meta-Analyses (PRISMA) diagram of search results. *Consecutive searches were automatically deduplicated against preceding results. †“Automation tools” on the Covidence platform were used to identify duplicates and non-RCT papers, and these were checked for accuracy. ‡5973 and 2282 exclusions based on clear and obvious information in the title indicating ineligibility for the review. §Not available in English. LTC=long term condition; RCT=randomised controlled trial

In total, we screened 8663 potentially relevant titles and abstracts, and assessed 407 full texts for eligibility, of which 349 were excluded, mainly because of an ineligible study population (n=291). We ultimately included 29 individual randomised controlled trials, reported in 43 papers (figure 1), comprising 9487 participants.^56^,^98^

### Study and intervention characteristics

Table 1 summarises the key characteristics of the 29 studies, and online supplemental table S4 describes all individual studies in detail. We identified 10 studies of organisational interventions (median duration 52 weeks), including six collaborative care, two stepped care, and two post-discharge interventions. Nineteen studies described patient oriented interventions (median duration 10 weeks), including six psychotherapy, four psychoeducation, and three exercise interventions. The comparator was usual care in 19 of the 29 studies, enhanced usual care in three studies (eg, training update for clinicians), and attention control in seven studies (eg, additional contact or reading materials).

**Table 1:** Summary of study characteristics

Study characteristic	Predominantly organisational interventions (n=10)	Predominantly patient oriented interventions (n=19)	All studies (n=29)
Intervention subtypes	Collaborative care: n=6 (60%)	Exercise: n=3 (16%)	—
	Post-discharge care: n=2 (20%)	Psychoeducation: n=4 (21%)	—
	Stepped care: n=2 (20%)	Psychotherapy: n=12 (63%)	—
Setting (No (%)):			
Primary care	7 (70)	3 (16)	10 (34)
Remote	0 (0)	6 (32)	6 (21)
Outpatient	1 (10)	4 (21)	5 (17)
Home	1 (10)	2 (11)	3 (10)
Mixed	1 (10)	2 (11)	3 (10)
Community venue	0 (0)	2 (11)	2 (7)
Mental health conditions included (No (%)):*			
Depression	7 (70)	12 (63)	19 (66)
Depression or anxiety	2 (20)	7 (37)	9 (31)
Subthreshold depression	1 (10)	0 (0)	1 (3)
Physical health conditions included (No (%)):			
Broad range of conditions	2 (20)	7 (37)	9 (31)
Diabetes and one other	6 (60)	5 (26)	11 (38)
Diabetes and two others	1 (10)	2 (11)	3 (10)
From one body system	1 (10)	5 (26)	6 (21)
Age inclusion criteria (No (%)):†			
Broad range of adults	8 (80)	11 (58)	19 (66)
Excluded younger adults	2 (20)	6 (32)	8 (28)
Excluded older adults	0 (0)	1 (5)	1 (3)
Excluded older and younger adults	0 (0)	1 (5)	1 (3)
Median of means (range of means) participants' age (years)‡	58 (55-77)	59 (38-80)	59 (38-80)
Median (range) duration (weeks)	52 (12-52)	10 (3-43)	12 (3-52)
Median (range) latest follow-up assessment (months)	12 (6-24)	6 (1-15)	6 (1-24)
Median (range) sample size	225 (46-2486)	201 (38-880)	211 (38-2486)
Geographical location (No (%)):			
Europe	4 (40)	9 (47)	13 (45)
North America	3 (30)	5 (26)	8 (28)
Asia	1 (10)	3 (16)	4 (14)
Australasia	2 (20)	1 (5)	3 (10)
South America	0 (0)	1 (5)	1 (3)
Risk of bias (No of studies):			
High	1	3	4
Some concerns	9	16	25
Low	0	0	0

Percentages may not add up to 100 due to rounding error.

* Mental health conditions listed in inclusion criteria of the trials.

† Online supplemental table S4 gives more details on age exclusions.

‡ Excludes two studies not reporting mean age of participants.

Twenty one studies were conducted in North America or Europe, most commonly in the US (n=5), UK (n=4), and the Netherlands (n=4). The earliest year of publication from the 29 studies was 2005, with 13 published since 2020. Nine studies (31%) excluded younger adults. Primary care was the most common setting (n=10, 34%). We observed heterogeneity in the socioeconomic characteristics of the study participants, and few studies focused predominantly on participants of low socioeconomic status. Two thirds of studies (n=10) had additional exclusion criteria relating to participants' age (ie, criteria other than adults being aged ≥18 years). Eight studies focused on older adults (most commonly adults aged ≥65 years, n=3), one study included participants aged 40-75 years, and one study restricted recruitment to people aged 18-55 years. Online supplemental table S4 describes the criteria of the individual studies.

### Risk of bias

Online supplemental figure S1 shows the results of the risk of bias assessments. We considered four studies to have an overall high risk of bias. In three of these studies, the risk of bias was based on a high risk rating in only one of the five domains in the RoB-V2 tool. In the fourth study, three out of the five domains were deemed high risk and the two other domains had “some concerns.” The remaining 25 studies each had some concerns in at least one domain and were therefore considered to have some risk of bias overall. The most common domain for risk of bias concerns was domain 2 (bias caused by deviation from the intended intervention), mainly because of the inability to mask participants to their intervention allocation.

Funnel plots showed marked asymmetry, suggesting small study bias (online supplemental figure S2). When the four studies with a high risk of bias were excluded, asymmetry was diminished and Egger's test was non-significant, suggesting an absence of publication bias in this subset.

### Intervention components

**Figure 2:**
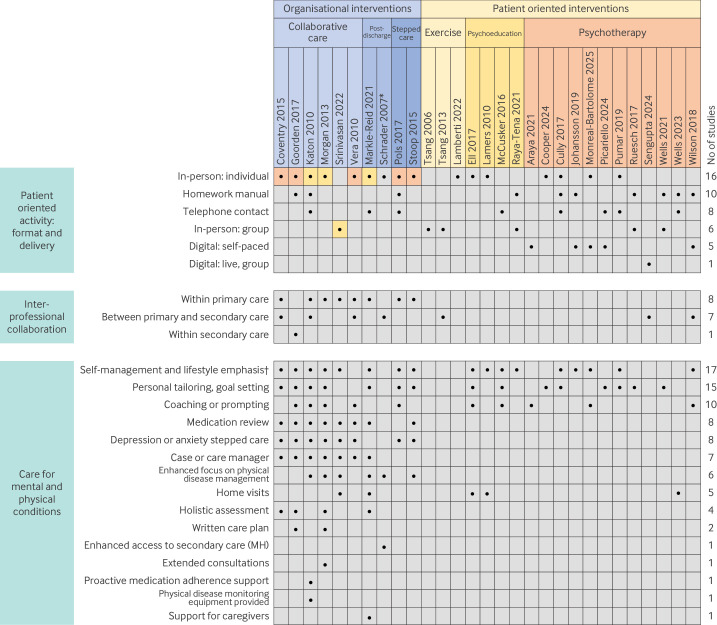
Matrix of intervention components. Colour in the study title indicates intervention subtype. Colour in the matrix for organisational interventions indicates what type of patient oriented activity was integrated into the intervention. *No colour given for Schrader 2007 as the only patient oriented activity was consultation (no exercise, psychoeducation or psychotherapy).†Although elements of self-management featured in many interventions, this was a dominant feature of the four psychoeducation interventions (see definitions in online supplemental box S2). MH=mental health

Figure 2 describes the components of each intervention. Patient oriented interventions typically involved one central component (exercise, psychotherapy, or psychoeducation) given in a variety of formats (eg, digital or in person, individual or group). Organisational interventions consisted of several components but typically integrated either psychotherapy or psychoeducation within a structured model of care. Across all 29 studies, 17 emphasised self-management and lifestyle, 15 included personal tailoring or goal setting, and 13 involved interprofessional communication. Interprofessional communication was a feature of all 10 organisational interventions, but only of three of the 19 patient oriented interventions. Structured reviews of drug treatment use featured in eight of the 10 organisational interventions, but in none of the patient oriented interventions. Self-management principles (as defined by the PRISMS taxonomy)^99^ were particularly prominent among all four psychoeducation interventions, which focused on increasing patients' understanding of depression and anxiety, the interplay of depression and anxiety with physical health, and the role of lifestyle and relaxation strategies (online supplemental box S2).

### Meta-analysis of intervention effects

**Figure 3:**
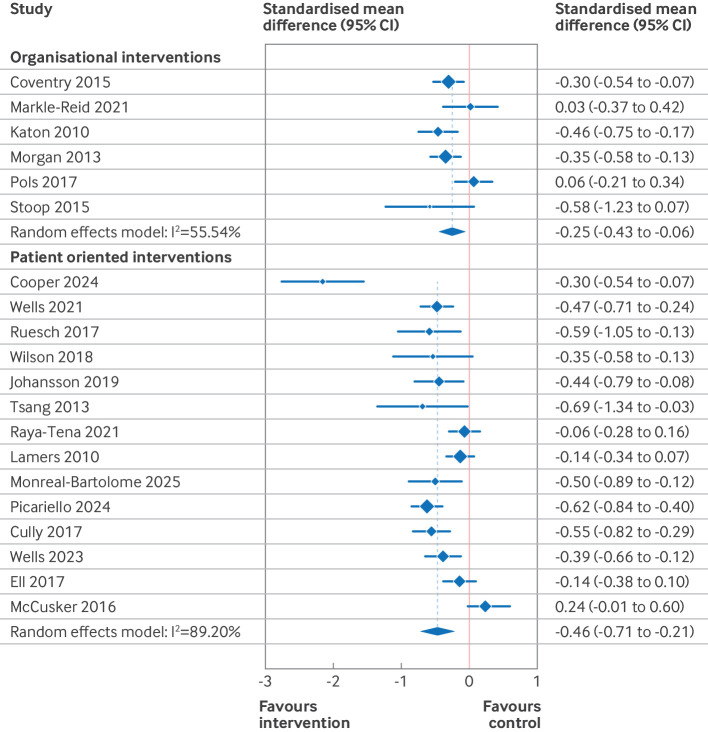
Forest plot for effect of organisational and patient oriented interventions on symptoms of depression at endpoint. Excludes studies with a high risk of bias. Within groups, studies are ordered by duration of intervention. CI=confidence interval

Based on the results of the risk of bias and funnel plot assessments, the primary results presented here (table 2, figures3^5^) are those of the predefined sensitivity analysis (excluding the studies with a high risk of bias). Online supplemental table S5 and figures S5-S11 give the results of the meta-analyses of all of the studies (including those with a high risk of bias). Table 2 presents the summary of findings from 11 separate meta-analyses, grouped into two intervention categories (organisational and patient oriented), three outcomes (depression, anxiety, and quality of life), and two time points (end intervention and late follow-up). For patient oriented interventions (median duration 10 weeks), we defined late follow-up as ≥12 months. For organisational interventions (median duration 52 weeks), late follow-up was defined as ≥18 months. Four of the 11 meta-analyses were based on high quality evidence (all organisational interventions), four were based on moderate quality of evidence (all patient oriented interventions), and three were either of low or very low quality.

**Table 2:** Summary of meta-analysis findings

Outcome	Assessment timing (range within group)	Effect of intervention on outcome	I^2^ (%)	No of participants (studies)	Quality of evidence*	Effect size	Summary
**Organisational interventions**							
Depression	End of intervention (4-12 months)	SMD 0.25 SD lower (0.43 lower to 0.06 lower)	55.54	1410 (6 RCTs)	High	Small	Organisational interventions result in a small reduction in depression at end intervention
	Late follow-up (18-24 months)	SMD 0.23 SD lower (0.53 lower to 0.08 higher)	68.85	883 (4 RCTs)	High	No significant effect	Organisational interventions result in little to no difference in depression at late follow-up
Anxiety	End of intervention (4-12 months)	SMD 0.11 SD lower (0.37 lower to 0.14 higher)	58.97	796 (4 RCTs)	High	No significant effect	Organisational interventions result in little to no difference in anxiety at end intervention
	Late follow-up (18-24 months)	SMD 0.32 SD lower (1.23 lower to 0.59 higher)	84.58	282 (2 RCTs)	Low a,b	No significant effect	Organisational interventions may result in little to no difference in anxiety at late follow-up
Quality of life	End of intervention (4-12 months)	SMD 0.21 SD higher (0.01 higher to 0.41 higher)	21.15	682 (3 RCTs)	High	Small	Organisational interventions result in a small improvement in quality of life at end intervention
	Late follow-up (18-24 months)	No studies reported data	—	—	—	—	No studies reported data
**Patient oriented interventions**							
Depression	End of intervention (3 weeks-6 months)	SMD 0.46 SD lower (0.71 lower to 0.21 lower)	89.20	2937 (14 RCTs)	Low b,c	Small	Patient oriented interventions probably result in a small reduction in depression at end-intervention
	Late follow-up (12 months)	SMD 0.17 SD lower (0.34 lower to 0.00 lower)	62.11	1723 (5 RCTs)	Moderate c	Trivial	Patient oriented interventions result in a trivial reduction in depression at late follow-up
Anxiety	End of intervention (3 weeks-4 months)	SMD 0.58 SD lower (0.98 lower to 0.17 lower)	93.38	1642 (7 RCTs)	Very low a,b,c	Moderate	Patient oriented interventions may result in a reduction in anxiety at end-intervention
	Late follow-up (12 months)	SMD 0.34 SD lower (0.48 lower to 0.21 lower)	0.00	995 (3 RCTs)	Moderate c	Small	Patient oriented interventions result in a small reduction in anxiety at late follow-up
Quality of life	End of intervention (3 weeks-4 months)	SMD 0.22 SD higher (0.14 higher to 0.29 higher)	0.00	2607 (9 RCTs)	Moderate c	Small	Patient oriented interventions result in a small improvement in quality of life at end-intervention
	Late follow-up (12 months)	SMD 0.15 SD higher (0.08 lower to 0.39 higher)	54.90	856 (3 RCTs)	Moderate c	No significant effect	Patient oriented interventions result in little to no difference in quality of life at late follow-up

Excludes studies with a high risk of bias.

Quality of evidence downgraded due to (a) because of: a=imprecision due to as a result of wide confidence interval (≥0.50 SD); (b) =substantial inconsistency (I2 ≥75%) in the pooled results; and (c) =indirectness in study design (eg, older adults only).

Effect size (SMD) thresholds: <0.2=trivial, ≥0.2=small, ≥0.5=moderate, and ≥0.8=large.

* Graded using the Grading of Recommendations Assessment, Development, and Evaluation framework

RCT, randomised controlled trial; SD, standard deviation; SMD, standardised mean difference.

**Figure 4:**
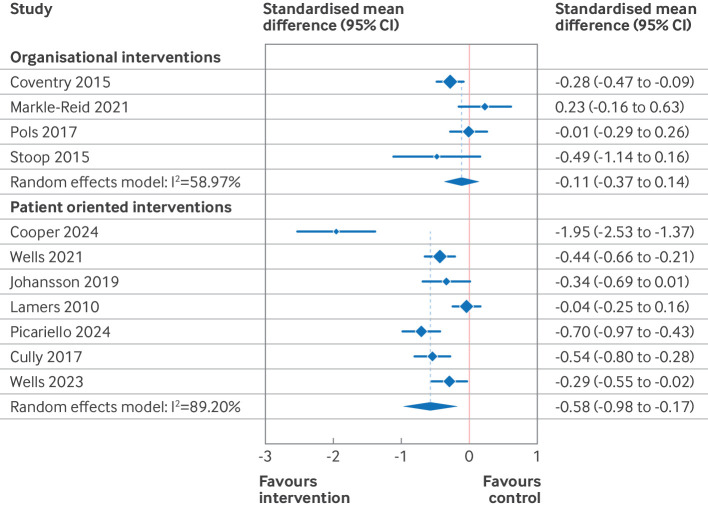
Forest plot for effect of organisational and patient oriented interventions on symptoms of anxiety at endpoint. Excludes studies with a high risk of bias. Within groups, studies are ordered by duration of intervention. CI=confidence interval

**Figure 5:**
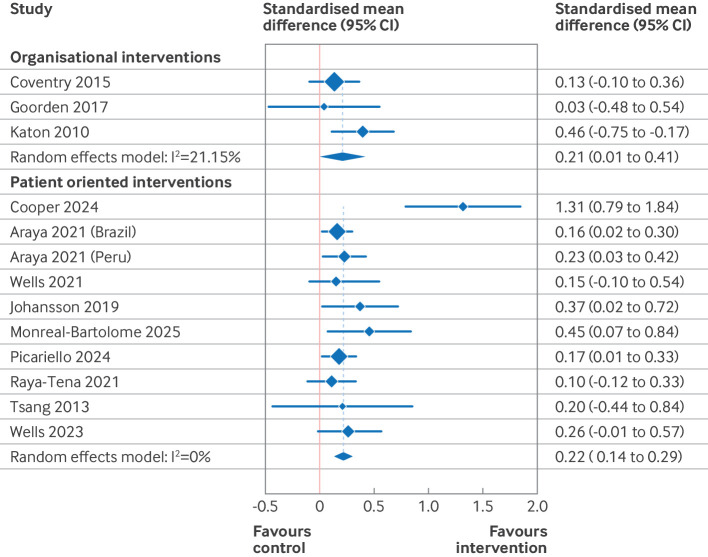
Forest plot for effect of organisational and patient oriented interventions on quality of life at endpoint. Excludes studies with a high risk of bias. Within groups, studies are ordered by duration of intervention. CI=confidence interval

We found high quality evidence that organisational interventions resulted in a significant small reduction in depressive symptoms at end intervention (standardised mean difference −0.25, 95% CI) −0.43 to −0.06, high quality evidence), but no significant difference at the late follow-up (standardised mean difference −0.23, −0.53 to 0.08, high quality evidence). For anxiety symptoms, we saw no effect at end intervention (standardised mean difference −0.11, 95% CI −0.37 to 0.14, high quality evidence) or at the late follow-up (−0.32, −1.23 to 0.59, low quality evidence). For quality of life, organisational interventions resulted in a small improvement at end intervention (standardised mean difference 0.21, 0.01 to 0.41, high quality evidence), and no studies reported data for late follow-up.

For patient oriented interventions, which were typically much shorter, we saw a small reduction in depressive symptoms and a moderate reduction in anxiety symptoms at end intervention (standardised mean difference −0.46, 95% CI −0.71 to −0.21, low quality evidence for depression; 0.58, −0.98 to −0.17, very low quality evidence for anxiety). For both outcomes, this effect was diminished at late follow-up (standardised mean difference −0.17, 95% CI−0.34 to 0.00, moderate quality evidence for depression; 0.34, −0.48 to −0.21, moderate quality evidence for anxiety). For quality of life, we saw a small improvement at end intervention (standardised mean difference 0.22, 0.14 to 0.29, moderate quality evidence), with little to no effect at late follow-up (0.15, −0.08 to 0.39, moderate quality evidence).

### Subgroup analysis

**Figure 6:**
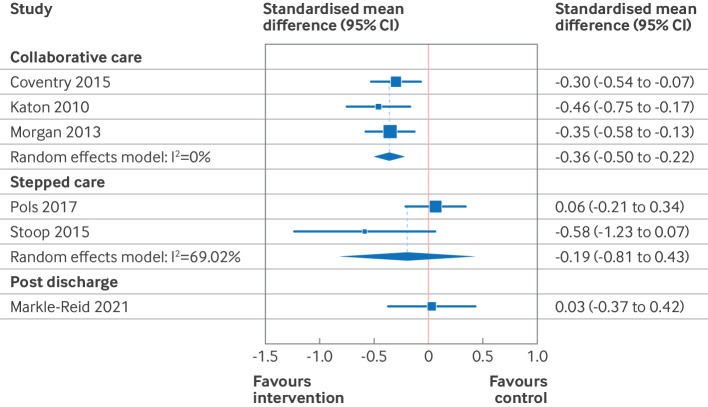
Forest plot for organisational intervention on symptoms of depression at 3-12 month. Excludes studies with a high risk of bias. Within groups, studies are ordered by duration of intervention. CI=confidence interval

The subgroup analysis (figures6  7 and online supplemental figure S4) grouped studies according to intervention subtype and used two consistent assessment time frames across all subgroups. In this analysis, we found that collaborative care and psychotherapy interventions both resulted in small reductions in depressive symptoms at 3-12 months after randomisation (standardised mean difference −0.36, 95% CI −0.50 to −0.22, high quality evidence for collaborative care; 0.38, −0.55 to −0.21, high quality evidence for psychotherapy). For collaborative care, these effects were sustained at 18-24 months (standardised mean difference −0.30, −0.50 to −0.22, high quality evidence), but no data were available for psychotherapy for this time frame. Collaborative care and psychotherapy also resulted in small and trivial (<0.2 standardised mean difference) improvements, respectively, in quality of life at 3-12 months (standardised mean difference 0.22, 0.03 to 0.42, high quality evidence for collaborative care; 0.17, 0.09 to 0.25, high quality evidence for psychotherapy). No data were reported for these subgroups for effect on quality of life at 18-24 months.

**Figure 7:**
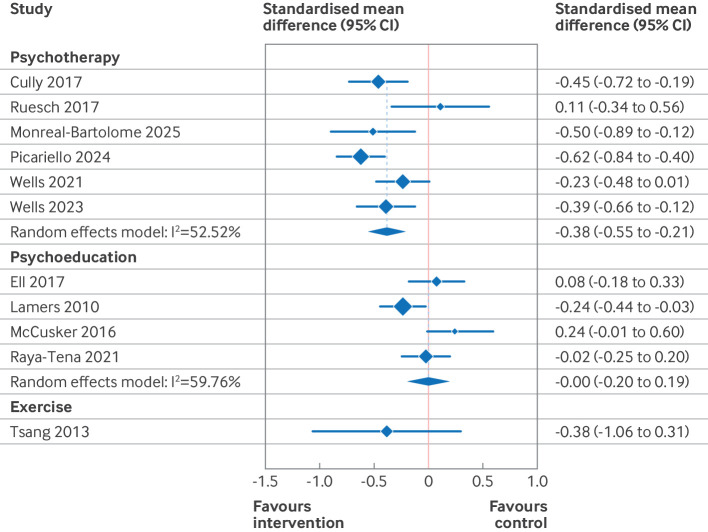
Forest plot for patient oriented intervention on symptoms of depression at 3-12 months. Excludes studies with a high risk of bias. Within groups, studies are ordered by duration of intervention. CI=confidence interval

### Synthesis without meta-analysis of effect on physical health outcomes

**Figure 8:**
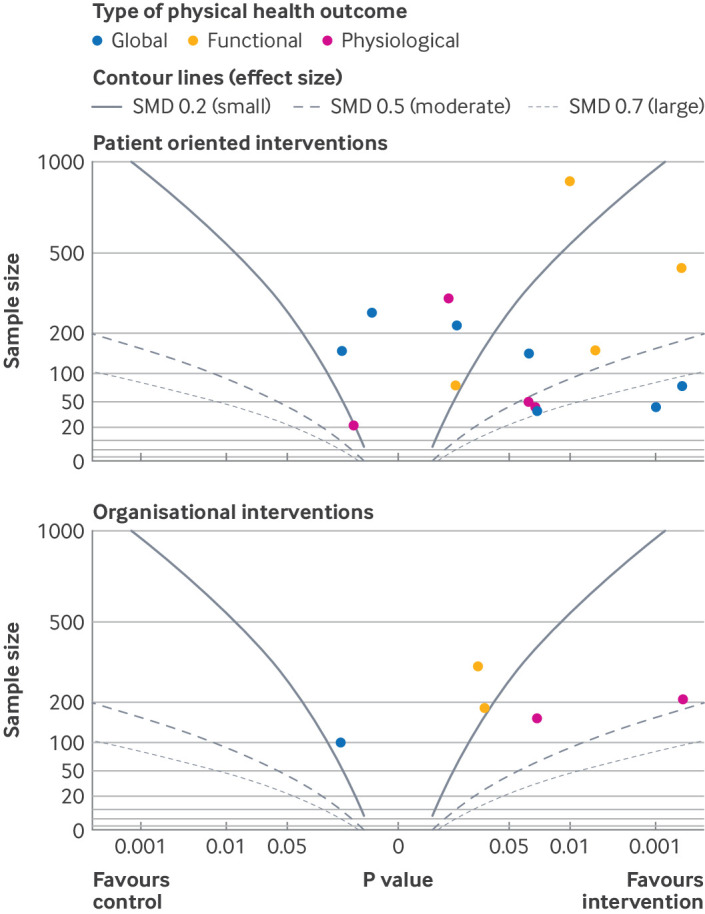
Albatross plots for effect of interventions on physical health outcomes. Excludes studies with a high risk of bias. SMD=standardised mean difference

Figure 8 (albatross plot) and online supplemental tables S7-S9 (Fisher's method for combining P values) show the results of the synthesis without meta-analysis for effect on physical health outcomes. The albatross plots show that for both organisational and patient oriented interventions, the results were generally clustered to the right, favouring the interventions. Heterogeneity among the studies was clear, however, particularly among the patient oriented interventions, with a wide range of study sizes (vertical spread) and P values (horizontal spread), and little consistency of results with respect to underlying effect size estimates (contour lines).

For patient oriented interventions, the most consistent pattern of results was for functional outcomes (eg, disability), with all four results centred to the right, clustered around the small effect size contour line (standardised mean difference 0.2). With Fisher's method for combining P values for this group, we found evidence of benefit in at least one study on physiological outcomes (P=0.02, four studies), functional outcomes (P<0.001, four studies) and global physical health outcomes (P<0.001, seven studies).

The albatross plot for organisational interventions showed that fewer than half (two out of five) of the reported results were significant (P<0.05). Both of these were for physiological outcomes, and both were within the small to moderate underlying effect size range. This finding is supported by Fisher's method for combining P values, which found evidence of benefit in at least one study on physiological outcomes (P<0.001, two studies, both collaborative care), but no evidence of benefit on functional outcomes (P=0.065, two studies, both collaborative care), and insufficient data to combine P values for global physical health outcomes (one study, post-discharge intervention).

## Discussion

### Principal findings

In this systematic review, we identified a wide range of interventions for people with multimorbidity who also had depression or anxiety. The interventions were categorised into two groups: predominantly organisational and predominantly patient oriented interventions. Organisational interventions had a median duration of 12 months and often integrated psychotherapy or psychoeducation within a structured model of care. We found high quality evidence that these interventions resulted in small improvements in depressive symptoms and quality of life at end intervention, but little or no difference in anxiety symptoms. At the late follow-up, the effects of organisational interventions on depressive and anxiety symptoms were not significant and no reported data were available for effect on quality of life. In the subgroup analysis, however, we found that collaborative care interventions resulted in a small sustained reduction in depression at 18-24 months after randomisation. Collaborative care was the only intervention subtype (across both organisational and patient oriented interventions) to show long term benefits. We also found evidence of benefit from organisational interventions (specifically collaborative care) on physiological outcomes (eg, haemoglobin A_1c_ levels), but not on functional (eg, disability) or global physical health outcomes.

Patient oriented interventions had a median duration of 10 weeks and typically had one central component: exercise, psychotherapy, or psychoeducation. We found low to moderate quality evidence that these interventions resulted in small improvements in depressive symptoms and quality of life, and very low quality evidence of moderate improvements in anxiety at end intervention. These benefits seemed to wane by late follow-up (12 months), and in subgroup analysis, we found no data to assess the long term effects of exercise, psychotherapy, or psychoeducation. We found evidence of benefit from patient oriented interventions (predominantly psychotherapy) on physiological, functional, and global physical health outcomes.

Almost half of the studies in this review (48%) specifically listed diabetes (with at least one other condition) in their physical condition inclusion criteria, including all six of the studies of collaborative care. This pattern reflects the particularly strong association between depression and diabetes that has been shown in observational studies,^100^ but may also indicate a degree of confirmation bias. More research is needed to examine whether interventions, such as collaborative care, also improve outcomes in more heterogenous populations with multimorbidity.

### Strengths and limitations of this study

The strengths of our study included the use of a comprehensive search strategy and robust analytical methods to identify, summarise, and synthesise the available evidence for a recognised research and policy priority (including meta-analysis and recommended methods for synthesis without meta-analysis, as appropriate).^101^ The grouping and component categorisation of interventions was based on well established taxonomies,^12 35 36^ allowing synthesis across studies with common features. Our main results were those of the prespecified sensitivity analyses, which excluded studies with a high risk of bias. One of the four studies excluded, which investigated a patient oriented intervention, was an outlier both in terms of its risk of bias assessment (with three out of five domains considered high risk) and in terms of results (with standardised mean difference improvements in depression, anxiety, and quality of life scores ranging from six to eight standard deviations).^89^ The results of the prespecified sensitivity analysis were therefore reported as primary. These results have both a higher degree of quality (because risk of bias was reduced, and precision and consistency were increased) and (for patient oriented interventions) smaller pooled effect estimates than the results of the meta-analyses of all of the studies originally prespecified as the primary analysis (online supplemental table S5).

In February 2026, an updated search by one reviewer found 1584 new titles with only one more eligible study identified.^102^ This study, conducted in Malawi, assessed an organisational intervention consisting of a tiered offering (stepped care model) of group problem solving therapy with or without the use of antidepressants. The study reported a moderate improvement in depressive symptoms (standardised mean difference −0.61, 95% CI −0.44 to −0.79), which is higher than the pooled estimate for organisational interventions found in our review. The study also reported significant improvements in systolic blood pressure and functional ability (WHO-Disability Assessment Schedule) which would have strengthened the evidence in the synthesised without meta-analysis of this review for physical health benefits from organisational interventions (at least in low and middle income countries).

Limitations of our study included heterogeneity in the physical conditions studied. By design, our review included only trials with more than one physical condition in their eligibility criteria so that we could identify interventions aimed at heterogenous populations with multimorbidity, as opposed to those studies tailored towards narrower populations with specific comorbidity disease pairs (eg, depression with cancer, or depression with diabetes). The actual conditions included, however, varied. For most studies, the list of physical conditions in the eligibility criteria covered multiple body systems, but six studies only included physical conditions within one body system (eg, only respiratory conditions^85^ or only dermatological conditions).^89^ Also, during the screening of search results, we noted that several potentially eligible studies were aimed at older populations with depression or anxiety, in whom the existence of (multiple) long term physical conditions was likely. These studies were excluded from the review, however, unless a long term physical condition, together with depression or anxiety, were explicitly stipulated as inclusion criteria. This approach may have led to the exclusion of evidence that was potentially relevant to the research question. Online supplemental table S10 gives an overview of the five studies excluded for this reason.

A further limitation was heterogeneity in the interventions included. The main analysis provided a broad comparison of organisational and patient oriented interventions, a grouping based on existing taxonomy and characterised by differences in intervention duration, intensity, and complexity. The differences in duration made comparing the effects of these two groups challenging and therefore two different approaches were adopted. The primary analysis examined the effects of two intervention groups at end intervention (determined by the intervention duration) and late follow-up (defined as ≥12 months after randomisation for patient oriented interventions and ≥18 months after randomisation for organisational interventions). Subgroup analysis then applied two consistent time frames (short term, 3-12 months after randomisation and long term, 18-24 months after randomisation) across all intervention subtypes, allowing for direct comparison at the cost of not incorporating evidence from studies not reporting within these time frames.

As well as challenges arising because of differences between these two groups of interventions, we found substantial heterogeneity within the two groups. For example, although psychological therapy, psychoeducation, and exercise interventions are patient oriented interventions, substantial differences exist in their modality, rationale, and mechanisms. These differences were reflected in the high levels of heterogeneity (I^2^ arising in the two group meta-analyses, which underscores the need for the subgroup analysis). The subgroup analysis, however, was itself limited by the small number of studies within each subgroup, meaning meta-analysis was not always possible. For example, only two exercise intervention studies provided suitable data for meta-analysis, and one of these had a high risk of bias. As a result, drawing conclusions about the effectiveness of exercise interventions for mental-physical multimorbidity was difficult, despite a strong evidence base in populations with multimorbidity and common mental disorders separately.^32 103^ A further source of heterogeneity was that studies were conducted in a range of health systems internationally, including some in low and middle income countries, with the type of usual care rarely explicitly reported, meaning that usual care cannot be easily compared between trials.

A final limitation was that our review meta-analysed the effects of interventions on depressive and anxiety symptoms reported as continuous outcomes, and not clinically defined depression or anxiety disorders, or dichotomous outcomes based on accepted cut-off values or minimal clinically important differences. Although our approach was typical for meta-analyses of randomised controlled trials, it could mean that the effect of an intervention on, for example, the Patient Health Questionnaire 9 item scale scores, did not lead to a participant dropping below the clinical threshold for depression. Improvements in depressive or anxiety symptoms are nevertheless worthwhile given their large societal burden and adverse effects on health.^104^,^106^ We could not group the meta-analysis by baseline symptom severity because these data were not consistently reported in individual studies.

### Comparison with other studies

Previous reviews have typically focused on specific types of interventions, such as collaborative care,^28 107^ psychological interventions,^27 108 109^ exercise,^32^ and self-management.^31^ Others have examined a wider range of interventions, but without a focus on mental-physical multimorbidity.^12^ Our review differed from these previous studies in its combination of an inclusive approach to intervention types (ie, spanning a range of organisational and patient oriented interventions), with a focused approach to study population (multimorbidity that included depression or anxiety, and where trial eligibility covered more than one physical condition).

Two previous reviews of psychological interventions using meta-analysis showed small to moderate improvements in both depression and anxiety symptoms in people with chronic physical conditions.^27 108^ Similarly, a review of self-management interventions found moderate effects on depression and anxiety at both early and late time points, as well as a small effect on physical health (glycaemic control).^31^ In each of these four reviews, most included studies stipulated only one physical comorbidity in their eligibility criteria, most often diabetes or cancer.

A review of exercise based interventions in people with multimorbidity (including only physical multimorbidity) showed a large effect on depressive symptoms (standardised mean difference −0.80) and smaller effects for physical function, quality of life, and anxiety.^32^ Thirteen of the 23 studies in the review included participants with multimorbidity involving depression, all of which stipulated one physical comorbidity (most commonly heart failure). Physical activity interventions are known to be beneficial across a range of populations, including older adults with depression,^103^ and can be combined with other components, such as self-management support.^110^

Smith et al identified 10 primary care or community interventions for people with multimorbidity, only two of which stipulated the presence of depression.^12^ This review found some evidence of benefit (based on narrative synthesis) from organisational interventions on depression, and none from patient oriented interventions, whereas evidence was mixed for physical health and quality of life outcomes.

Collaborative care was found to be beneficial in people with mental-physical multimorbidity in a recent review based on narrative synthesis,^28^ as well as in a previous review based on a meta-analysis that included people with depression or anxiety problems (with or without coexisting physical health conditions).^107^ In the latter study, the small effects on depressive and anxiety symptoms were sustained at late follow-up, consistent with the findings of the subgroup analysis in our review. This review therefore adds to an existing evidence base for the effectiveness of collaborative care, particularly by showing an effect on both quality of life and mental health in people with mental-physical multimorbidity. Originally developed in the US over 20 years ago as a model for managing depression in primary care,^111^ collaborative care is an organisational intervention typically with a multi-professional team (mental health professional, general practitioner, and case manager), enhanced interprofessional communication (regular meetings or shared records), and a structured and patient centred management plan including evidence based treatments (such as psychological therapy together with physical health and drug treatment reviews).^112^ Despite its strong evidence base, several barriers to real world implementation of collaborative care have been identified, including the complexity of organisational redesign required.^112 113^

### Implications for policy, practice, and research

Despite the small effect sizes found for the interventions in this review, the prevalence and impact of multimorbidity involving depression or anxiety means that the potential benefits of large scale implementation are still substantial. The findings of this review are therefore relevant to healthcare providers who, alongside the findings of this study, should consider the cultural suitability of interventions, availability of resources, and the degree of system reorganisation required within their settings. Future research should explore possible ways that the implementation of effective but complex interventions, such as collaborative care, can be supported within primary care systems. Collaborative care was the only intervention to show long term benefits in this review and has been recommended as a framework for integrating mental and physical healthcare in low and middle income countries,^114^ as well as in clinical guidelines for the management of depression with co-existing physical conditions in the UK and US.^33 115^

In contrast, psychotherapy interventions, which can be delivered in online and group formats, may offer a more feasible and inexpensive alternative to organisational interventions, while having similar short term benefits. Future research should therefore explore how the early benefits of patient orientated interventions might be sustained, including through the use of artificial intelligence driven conversational agents that are increasingly being used for healthcare (particularly mental health), although uncertainties over their safety and effectiveness remain.^116^

Our review highlighted the scarcity of trials of exercise interventions in people with mental-physical multimorbidity, despite evidence of their impact on mental health outcomes in a range of populations. Future research should look at this gap. Furthermore, although most studies reported indicators of participants' socioeconomic position, few interventions focused predominantly on participants from socioeconomically deprived groups. Greater focus on this population in future research would be warranted given the high prevalence of mental-physical multimorbidity in people from poorer socioeconomic groups, especially adults of working age.^1^ Finally, the high proportion of studies (particularly of collaborative care) which listed diabetes with one other condition in their physical condition inclusion criteria highlights the need to consider whether such interventions are effective in more heterogenous populations with multimorbidity, as well as whether certain clusters of mental-physical multimorbidity are more responsive to certain interventions.

## Conclusions

Improving key outcomes among heterogenous populations with multimorbidity who also have depression or anxiety is possible, but the evidence suggests that the effect of existing interventions is small and, for patient oriented interventions in particular, diminishes over time. Future research is needed to improve the management of multimorbidity involving depression or anxiety, which has a substantial effect on individuals, systems, and society.

## Supplementary material

10.1136/bmjmed-2025-002400 online supplemental file 1

10.1136/bmjmed-2025-002400 online supplemental file 2

10.1136/bmjmed-2025-002400 online supplemental file 3

## Data Availability

No data are available.
