# Supplementary material for: Primary care and community interventions for multimorbidity involving depression or anxiety: systematic review with meta-analysis
Source: BMJ Med. 2026 Apr 10;5(1):e002400. doi: 10.1136/bmjmed-2025-002400 (PMC13084858; doi:10.1136/bmjmed-2025-002400)
Supplement: online supplemental file 1 [file bmjmed-5-1-s001.pdf]

Interventions for mental-physical multimorbidity: A systematic review

Kieran Sweeney, Bruce Guthrie, Lucy Stirland, Atul Anand, Clare MacRae, Michaela Gilarova, Lauren Ng, Susanne Maxwell, Jennifer Baker

Citation
Kieran Sweeney, Bruce Guthrie, Lucy Stirland, Atul Anand, Clare MacRae, Michaela Gilarova, Lauren Ng, Susanne Maxwell, Jennifer Baker. Interventions for mental-physical multimorbidity: A systematic review. PROSPERO 2025 CRD420251004355. Available from https://www.crd.york.ac.uk/PROSPERO/view/CRD420251004355.

REVIEW TITLE AND BASIC DETAILS

Review title

Interventions for mental-physical multimorbidity: A systematic review

Condition or domain being studied

Mental-physical multimorbidity involving depression and/or anxiety alongside different physical long-term conditions.

Rationale for the review

Mental-physical multimorbidity refers to the coexistence of mental and physical long-term conditions. The most prevalent type of mental-physical multimorbidity is that involving common mental disorders such as depression or anxiety. When mental and physical conditions coexist, they often complicate each other's management and can have a significant impact on individuals' quality of life. Mental-physical multimorbidity is much more common in areas of high deprivation and is associated with increased utilization of health services. Improving outcomes for people with mental-physical multimorbidity is a priority for both patients and policy makers.

Previous reviews of evidence for interventions targeting mental-physical multimorbidity have focussed on particular types of interventions, or have looked at common mental disorders coexisting with single index conditions, such as diabetes. The aim of this review is to examine the evidence for any interventions targeting people with mental-physical multimorbidity, where that involves common mental disorders alongside different long-term physical conditions.

Review objectives

Research Questions

1. What interventions are effective for adults with mental-physical multimorbidity where that involves depression and/or anxiety alongside different long-term physical conditions?
2. What are the components which make up these interventions, and how are they delivered?

Keywords
Multimorbidity; Mental Health; Interventions

Country
Scotland

ELIGIBILITY CRITERIA

Population

Included
The condition of interest is mental-physical multimorbidity involving depression and/or anxiety alongside different long-term physical conditions. Trial participants must have a diagnosis or symptoms of depression and/or anxiety, as well as at least one long-term physical condition from a range of two or more physical conditions in the trial's eligibility criteria.

In addition, the study population will be limited to adult participants aged 18 years and over.

Excluded
Trials whose participants must have a single, index physical condition are excluded from this review. Trials targeting peri/post-natal depression or other mental disorders such as psychotic disorders, bipolar depression, obsessive compulsive disorder, neurodevelopmental disorders, alcohol and drug misuse and dementia are also excluded from this review (including when other mental disorders co-exist with depression and/or anxiety).

Intervention(s) or exposure(s)

Included
Interventions may include any strategies or models of care addressed to patients in the defined population.

Excluded
Medication-only interventions, inpatient interventions, procedural interventions and interventions aimed only at carer-givers will be excluded.

Comparator(s) or control(s)

Included
Included studies will compare interventions to no treatment, usual care or to an attention control.

Study design
Only randomized study types will be included.

Included
The review will include studies of randomized control trials (RCTs). Pragmatic RCTs and pilot RCTs with 30 or more participants will also be included. No date restrictions will be applied. Studies published up to the date of the search will be included.

Excluded

Studies not published in English will be excluded. Pilot or feasibility studies with fewer than 30 participants, single arm pre-post trials, prevention trials, systematic reviews, meta-analyses, study protocols, case studies and comment articles or similar will be excluded.

Context

The setting for interventions must be primary care, ambulatory care or a community setting. Inpatient interventions, and interventions set within day-hospital services such as dialysis or chemotherapy units will be excluded.

TIMELINE OF THE REVIEW

Date of first submission to PROSPERO

04 March 2025

Review timeline

Start date: 4 March 2025. End date: 4 September 2025.

Date of registration in PROSPERO

07 April 2025

AVAILABILITY OF FULL PROTOCOL

Availability of full protocol

A full protocol has been written and uploaded to PROSPERO. The protocol may be accessed through this link https://www.crd.york.ac.uk/PROSPERO/PROSPEROFILES/f8d16a67c156f30e94a6c14a188ed362.pdf.

SEARCHING AND SCREENING

Search for unpublished studies

Only published studies will be sought.

Main bibliographic databases that will be searched

The main databases to be searched are *CENTRAL - Cochrane Central Register of Controlled Trials*, *CINAHL - Cumulative Index to Nursing and Allied Health Literature*, *Embase - Embase via Ovid*, *MEDLINE* and *PsycInfo*.

Other important or specialist databases that will be searched
Web of Science

Search language restrictions

The review will only include studies published in English.

Search date restrictions

There are no search date restrictions.

Other methods of identifying studies

Other studies will be identified by: *looking through all the articles that cite the papers included in the review ("snowballing")* and *reference list checking*.

Addiitonal information about identifying studies

References from any relevant systematic reviews identified will also be hand-searched.

Link to search strategy

A full search strategy has been uploaded to PROSPERO. The PDF may be accessed through this link https://www.crd.york.ac.uk/PROSPERO/PROSPEROFILES/8595e4aabe99cbdad59e85c2034417fa.pdf.

Selection process

Studies will be screened independently by at least two people (or person/machine combination) with a process to resolve differences.

Other relevant information about searching and screening

None

DATA COLLECTION PROCESS

Data extraction from published articles and reports

Data will be extracted by one person (or a machine) and checked by at least one other person (or machine).

Authors will be asked to provide any required data not available in published reports.

Study risk of bias or quality assessment

Risk of bias will be assessed using: *Cochrane RoB-2*

Data will be assessed by one person (or a machine) and checked by at least one other person (or machine).

Additional information will **not** be sought from study investigators if required information is unclear or unavailable in the study publications/reports.

Reporting bias assessment

Risk of bias due to missing results will not be assessed

Certainty assessment

Overall quality of evidence will be assessed using GRADE criteria.

OUTCOMES TO BE ANALYSED

Main outcomes

Mental health outcomes, quality of life outcomes, physical health outcomes.

Additional outcomes

Other patients reported outcomes or behaviours including adherence, self-efficacy and treatment burden.

PLANNED DATA SYNTHESIS

Strategy for data synthesis

A structured descriptive comparison of study interventions, populations and results will be presented. Meta-analysis of study results will be conducted, if meaningful and appropriate to do so, calculating pooled estimates of effects for similar outcomes (standardized mean difference for

continuous outcomes, odds ratios for dichotomous outcomes). Sensitivity analysis will exclude those with high risk of bias. Heterogeneity among studies will be calculated using I2. Subgroup analysis will be performed, if appropriate and feasible, according to setting and intervention type. Where meta-analysis is not possible, narrative synthesis of results will be conducted, with simple descriptive quantification across studies. Descriptive synthesis of components of interventions will be based on the Cochrane Effective Practice and Organisation of Care (EPOC) taxonomy of health systems interventions,(7) and the National Institute for Health and Care Excellence (NICE) multimorbidity guideline document.(8)

CURRENT REVIEW STAGE

Stage of the review at this submission

Review stage	Started	Completed
Pilot work	✓	✓
Formal searching/study identification	✓	✓
Screening search results against inclusion criteria	✓	
Data extraction or receipt of IPD		
Risk of bias/quality assessment		
Data synthesis		

Review status
The review is currently planned or ongoing.

Publication of review results
Results of the review will be published.

REVIEW AFFILIATION, FUNDING AND PEER REVIEW

Review team members
Dr Kieran Sweeney (review guarantor and contact) The University of Edinburgh. Scotland.

No conflict of interest declared.

Professor Bruce Guthrie. University of Edinburgh. Scotland.

No conflict of interest declared.

Dr Lucy Stirland. The University of Edinburgh. Scotland.

No conflict of interest declared.

Dr Atul Anand. University of Edinburgh. Scotland.

No conflict of interest declared.

Dr Clare MacRae. University of Edinburgh. Scotland.

No conflict of interest declared.

Ms Michaela Gilarova. University of Edinburgh. Scotland.

No conflict of interest declared.

Dr Lauren Ng. University of Edinburgh. Scotland.

No conflict of interest declared.

Dr Susanne Maxwell. University of Edinburgh. Scotland.

No conflict of interest declared.

Dr Jennifer Baker. NHS Education Scotland. Scotland.

No conflict of interest declared.

Named contact

Dr Kieran Sweeney (ksweeney@ed.ac.uk). The University of Edinburgh. Scotland.

Review affiliation

University of Edinburgh

Funding source

Wellcome Trust.

Grant number
Reference 223499/Z/21/Z

Additional non-commercial funding information
Wellcome Trust Multimorbidity PhD Programme for Healthcare Professionals. Reference 223499/Z/21/Z

Peer review

There has been no peer review of this planned review.

ADDITIONAL INFORMATION

Additional information

References

1. Banstola A, Pokhrel S, Hayhoe B, Nicholls D, Harris M, Anokye N. Economic evaluations of interventional opportunities for the management of mental–physical multimorbidity: a systematic review. BMJ Open. 2023;13(2):e069270.

2. Cimpean D, Drake RE. Treating co-morbid chronic medical conditions and anxiety/depression. Epidemiology and Psychiatric Sciences. 2011;20(2):141-50.

3. Smith SM, Wallace E, Clyne B, Boland F, Fortin M. Interventions for improving outcomes in patients with multimorbidity in primary care and community setting: a systematic review. Syst Rev. 2021;10(1):271.

4. Lammila-Escalera E, Greenfield G, Pan Z, Nicholls D, Majeed A, Hayhoe B. Interventions to improve medication adherence in adults with mental–physical multimorbidity in primary care: a systematic review. Br J Gen Pract. 2024:BJGP.2023.0406.

5. Smith SM, Wallace E, Salisbury C, Sasseville M, Bayliss E, Fortin M. A Core Outcome Set for Multimorbidity Research (COSmm). The Annals of Family Medicine. 2018;16(2):132-8.

6. Higgins JPT SJ, Page MJ, Elbers RG, Sterne JAC. Chapter 8: Assessing risk of bias in a randomized trial. In: Higgins JPT TJ, Chandler J, Cumpston M, Li T, Page MJ, Welch VA, editor. Cochrane Handbook for Systematic Reviews of Interventions Cochrane2024.

7. Cochrane Collaboration Effective Practice and Organisation of Care (EPOC). EPOC Taxonomy. 2021 [Accessed 27.02.25]. Available from: https://doi.org/10.5281/zenodo.5105851

8. Multimorbidity: clinical assessment and management. NICE guideline NG56. National Institute for Health and Care Excellence2016 [Accessed 27.02.25]. Available from: https://www.nice.org.uk/guidance/ng56.

Review conflict of interest

Declared individual interests are recorded under team member details.. No additional interests are recorded for this review.

Medical Subject Headings

Multimorbidity; Depression; Anxiety Disorders

SIMILAR REVIEWS

Check for similar records already in PROSPERO

PROSPERO identified a number of existing PROSPERO records that were similar to this one (last check made on 4 March 2025). These are shown below along with the reasons given by that the review team for the reviews being different and/or proceeding.

- Psychosocial interventions to prevent anxiety and depression in inflammatory rheumatological and other long term conditions: a systematic review [published 26 May 2022] [CRD42022333954]. The review was judged **not to be similar**
- The role of primary care in reducing the decline in physical function and physical activity in people with long-term conditions; what works, for whom and in what circumstances? A realist synthesis of evidence [published 30 August 2018] [CRD42018103027]. The review was judged **not to be similar**
- Mindfulness-Based Interventions for Wellbeing and Mental Health in Children and Adolescents: A Systematic Review [published 8 July 2024] [CRD42024539526]. The review was judged **not to be similar**

PROSPERO version history

- Version 1.0, published 07 Apr 2025

Disclaimer

The content of this record displays the information provided by the review team. PROSPERO does not peer review registration records or endorse their content.

PROSPERO accepts and posts the information provided in good faith; responsibility for record content rests with the review team. The guarantor for this record has affirmed that the information provided is truthful and that they understand that deliberate provision of inaccurate information may be construed as scientific misconduct.

PROSPERO does not accept any liability for the content provided in this record or for its use. Readers use the information provided in this record at their own risk.

Any enquiries about the record should be referred to the named review contact
