# Supplementary material for: Primary care and community interventions for multimorbidity involving depression or anxiety: systematic review with meta-analysis
Source: BMJ Med. 2026 Apr 10;5(1):e002400. doi: 10.1136/bmjmed-2025-002400 (PMC13084858; doi:10.1136/bmjmed-2025-002400)
Supplement: online supplemental file 3 [file bmjmed-5-1-s003.pdf]

Primary care and community interventions for multimorbidity involving depression or anxiety: A systematic review with meta-analysis

Supplementary Material

Kieran Sweeney, Michaela Gilarova, Lauren Ng, Jennifer Baker, Susanne Maxwell, Clare MacRae, Stewart W Mercer, Atul Anand, Bruce Guthrie, Lucy E Stirland

Table of Contents

Box S1. Search terms used ... 2

Table S1. Abbreviations used for quality of life and wellbeing outcome measures ... 3

Table S2. Abbreviations used for depression and anxiety outcome measures ... 3

Table S3. Abbreviations used for physical health outcome measures ... 4

Box S2. Categorisation of intervention subtypes ... 5

Table S4. Key characteristics of each study ... 6

Figure S1. Risk of bias assessment ... 12

Figure S2. Funnel plots for synthesis groups with ten or more studies ... 13

Table S5. Summary of findings of meta-analysis for all studies including high risk of bias. ... 14

Table S6. Summary of findings of subgroup meta-analysis (effects on intervention subtypes on depression, anxiety and quality of life, excluding high risk of bias studies) ... 15

Figure S3. Meta-analysis of effect of interventions on depressive symptoms at end-intervention and late follow-up. ... 17

Figure S4. Meta-analysis of effect of intervention subtypes on quality of life at 3-12 months ... 18

Figure S5. Effect of organisational interventions on anxiety at late follow-up (18-24m) ... 19

Figure S6. Effect of patient-level interventions on anxiety at end-intervention (3wk – 6m), including high risk of bias studies (red dot) ... 20

Figure S7. Effect of patient-level interventions on anxiety at late follow-up (12m) ... 20

Figure S8. Effect of organisational interventions on depression at end-intervention (4-12m), including high risk of bias studies (red dot) ... 21

Figure S9. Effect of patient-level interventions on quality of life at end-intervention (3wk – 6m), including high risk of bias studies (red dot) ... 21

Figure S10. Effect of patient-level interventions on quality of life at late follow-up (12m) ... 22

Figure S11. Effect of patient-level interventions on depression at end-intervention (3wk – 6m), including high risk of bias studies (red dot) ... 22

Table S7. Effects on physical health outcomes, grouped as global, functional and physiological ... 23

Table S8. Results of Fisher's method for combining p-values by intervention group, including high risk of bias studies ... 24

Table S9. Results of Fisher's method for combining p-values by intervention subtype, including high risk of bias studies ... 24

Box S3. Reasons for exclusion from meta-analysis ... 25

Table S10. Overview of studies in older populations with depression where the presence of a physical long-term conditions was not stipulated. ... 25

Box S1. Search terms used

Concept	Search	OVID (MEDLINE)
	1	Comorbidity/ or Multimorbidity/
	2	multiple long term conditions.ti,ab.
	3	(comorbid* or co-morbid*).ti,ab.
	4	(multimorbid* or multi-morbid*).ti,ab.
	5	((coexisting or co-existing or co-occurring or concurrent or multi or multiple) adj2 (ill* or disease? or condition?)).ti,ab.
MLTC:	6	or/1-5
	7	Chronic Disease/
	8	((long term or chronic) adj2 (ill* or disease? or condition?)).ti,ab.
	9	(cardiovascular disease or respiratory disorder? or chronic obstructive pulmonary disorder or asthma or diabetes or cardiac or coronary artery disease or coronary heart disease or heart failure or CVD or chronic respiratory disease or COPD or cancer? or epilepsy or stroke or multiple sclerosis or parkinson? or neurodegenerative condition? or arthritis or inflammatory arthropath? or liver disease or cirrhosis or HIV or AIDS or human immunodeficiency virus or acquired immunodeficiency syndrome).ti.
LTC:	10	or/7-9
	11	anxiety disorders/ or mood disorders/ or depressive disorder/
	12	(mental adj2 physical).ti.
	13	(depress* or anxiety or mood disorder? or low mood or common mental disorder?).ti.
MH:	14	or/11-13
MPM:	15	(6 or 10) and 14
	16	(treatment or management or intervention or program$).ti.
	17	(exercise? or rehab$ or physical activity or physical therapy or physiotherapy or aerobic training or psychological therapy or psychotherapy or self management or self care).ti,ab.
	18	((collaborative or coordinated or co-ordinated or integrated or chronic) adj care).ti,ab.
	19	(patient adj2 educat*).ti,ab.
INT:	20	or/16-19
	21	exp randomized controlled trial/
	22	controlled clinical trial.pt.
	23	randomi#ed.ab.
	24	clinical trials as topic/
	25	randomly.ab.
	26	trial.ti.
RCT:	27	or/21-26
	28	exp animals/ not humans/
	29	(review or meta analysis or news or comment or editorial).pt.
	30	cochrane database of systematic reviews.jn.
	31	comment on.cm.
	32	(systematic review or literature review or protocol).ti.
EXCL:	33	or/28-32
	34	27 not 33
	35	15 and 20 and 34
ALL:	36	limit 35 to english

These search terms, initially designed for OVID MEDLINE, were translated for other databases (EMBASE, Cochrane Library, CINAHL, PsycInfo and Web of Science) using the Polyglot Search Translator developed by Bond University, Australia.[1]

Table S1. Abbreviations used for quality of life and wellbeing outcome measures

Abbreviation	Full Name	Construct Measured
EQ-5D-3L / 5L	EuroQol 5-Dimensions 3-level / 5-level versions	Health-related quality of life
EQ VAS	EuroQol Visual Analogue Scale	Self-rated health
FI	Flourishing Index	Wellbeing
SF-12 / 36	Short Form Health Survey 12-item / 36-item versions	Health-related quality of life
SQCPD Personal Wellbeing Subscale	Self-concept Questionnaire for Hong Kong Chinese Adults with Physical Disabilities	Wellbeing
VR 12	Veterans RAND 12-item Health Survey	Health-related quality of life
WHO Wellbeing Index	World Health Organization Wellbeing Index	Wellbeing
WHO-QOL BREF	World Health Organization Quality of Life Instrument Short Form	Quality of life

Table S2. Abbreviations used for depression and anxiety outcome measures

Abbreviation	Full Name	Construct Measured
BAI	Beck Anxiety Inventory	Anxiety
DASS-A	Depression Anxiety Stress Scales - Anxiety Subscale	Anxiety
GAD-7	Generalized Anxiety Disorder 7-item scale	Anxiety
GAI	Geriatric Anxiety Inventory	Anxiety (older adults)
HADS-A	Hospital Anxiety and Depression Scale - Anxiety Subscale	Anxiety
BDI	Beck Depression Inventory	Depression
CES-D-10	Center for Epidemiologic Studies Depression Scale 10-item	Depression
DASS-D	Depression Anxiety Stress Scales - Depression Subscale	Depression
GDS	Geriatric Depression Scale	Depression (older adults)
HADS-D	Hospital Anxiety and Depression Scale - Depression Subscale	Depression
HRDS	Hamilton Rating Scale for Depression	Depression
MADRS-S	Montgomery–Åsberg Depression Rating Scale - Self-report version	Depression
PHQ-9	Patient Health Questionnaire 9-item scale	Depression
SCL-D13, SCL-D20	Symptom Checklist, 13-item/20-item	Depression

Table S3. Abbreviations used for physical health outcome measures

Abbreviation	Full Name	Construct Group
PGI-S	Patient Global Impression - Severity	Global physical health rating
SF-12/36-PCS	Short Form Health Survey – 12-item / 36-item versions - Physical Component Score	Global physical health rating
SQCPD Physical Wellbeing Subscale	Self-concept Questionnaire for Hong Kong Chinese Adults with Physical Disabilities	Global physical health rating
VR-12-PCS	Veterans RAND 12-item Health Survey - Physical Component Summary	Global physical health rating
FRS-R	Functional Rating Scale - Revised	Functional
RMD-Q	Roland-Morris Disability Questionnaire	Functional
SDS	Sheehan Disability Scale	Functional
WHO-DAS	World Health Organization Disability Assessment Schedule	Functional
WSAS	Work and Social Adjustment Scale	Functional
BMI	Body Mass Index	Physiological
HbA1c	Haemoglobin A1c	Physiological
SBP	Systolic Blood Pressure	Physiological

Box S2. Categorisation of intervention subtypes

**Patient-oriented interventions**<br>Interventions directed primarily at individuals and typically involving one central component.[2]	
Exercise	Interventions involving a structured programme of supervised or unsupervised physical activity, such as aerobic exercise, strength training or flexibility training, undertaken to improve health outcomes.[3]
Psychoeducation	A programme of training or information delivery aimed at patients with mental disorders which aims to increase their understanding of a condition, its symptoms and management, particularly self-management and lifestyle advice.[4]
Psychotherapy	A course of structured therapeutic conversations or interactions, based on a defined conceptual model with a specified methodology and focus, and aiming to treat mental disorders or psychological problems. Common examples include cognitive behavioural therapy, psychodynamic therapy and interpersonal therapy.[5]
**Organisational interventions**<br>Interventions that involve changes to the delivery of care in order to better meet patients' needs, for example through care coordination or the involvement of other health professionals. These interventions typically consisted of multiple components (which could include patient-oriented interventions) within a structured model of care.[2]	
Collaborative care	A healthcare system–level intervention, based on principles of chronic disease management, involving: [1] a multiprofessional team including a case/care manager, a primary care or general physician and mental health specialist; [2] enhanced interprofessional communication (e.g., regular meetings or shared records); [3] a structured, patient-centred management often including psychological therapy or education alongside scheduled physical health and medication reviews.[6]
Stepped-care	A hierarchical model of care, where patients initially receive the least intensive care appropriate to their concerns, and those who do not respond are 'stepped up' to consecutively more intensive interventions at scheduled follow-up reviews.[7]
Post-discharge	A type of transitional care intervention specifically focussing on the period following a patient's discharge from hospital to the community. Components often include follow up reviews, self-management support and enhanced interprofessional communication.[8]

Table S4. Key characteristics of each study

Study, Country	Eligibility criteria<br>- Age<br>- Mental condition(s)<br>- Physical conditions	Participants:<br>- Sample size (n)<br>- Mean age<br>- % female<br>- Socioeconomic status	Setting	Intervention group:<br>- Intervention subtype<br>(Detail)	Comparator	Duration (D) and Follow-up (FU)	Risk of bias	Mental health outcomes	Quality of life (QoL) outcomes	Physical health outcomes<br>- Global (G)<br>- Functional (F)<br>- Physiological (P)
ORGANISATIONAL INTERVENTIONS: COLLABORATIVE CARE										
Coventry 2015, UK	Age: 18+ years<br>Depression (PHQ9≥10)<br>DM, CHD	n = 387<br>Mean age: 58.7 years<br>38% female<br>SEP: 32% high deprivation (of three levels)	Primary care	Organisational:<br>- Collaborative care	Usual care	(D) 12 weeks<br>(FU) 4, 24 months	Some	SCL-D13⁼<br>PHQ-9<br>GAD-7	WHO-QOL-BREF	(F) SDS+
Goorden 2017, Netherlands	Age 18+ years<br>Depression (MDD)<br>DM, COPD, HF, CHD	n = 81<br>Mean age: 58.4 years<br>39% female<br>SEP: 28% education beyond high school	Outpatient clinic (medical)	Organisational:<br>- Collaborative care	Usual care	(D) 22 weeks<br>(FU) 3, 6, 9, 12 months	Some	None	EQ-5D-3L	None
Katon 2010, USA	Age 18+ years<br>Depression (PHQ9≥10)<br>DM, CHD	n = 214<br>Mean age: 56.8 years<br>52% female<br>SEP: 58% college education	Primary care	Organisational:<br>- Collaborative care	Usual care	(D) 12 months<br>(FU) 6, 12, 18, 24 months	Some	SCL-D20	Single item global QoL rating	(F) WHO-DAS+, SDS<br>(P) Composite of HbA1c, SBP, LDL
Morgan 2013, Australia	Age 18+ years<br>Depression (PHQ9≥5)<br>DM, CHD	n = 400<br>Mean age: 67.8 years<br>SEP: 47% female<br>SEP: NR	Primary care	Organisational:<br>- Collaborative care	Usual care	(D) 12 months<br>(FU) 6 months	Some	PHQ-9	None	(P) HbA1c+, BMI, SBP, lipids
Srinivasan 2022, India	Age 30+ years<br>Depression or anxiety (MINI)<br>DM, CHD, HTN	n = 2486<br>Mean age: 59.2 years<br>75% female<br>SEP: 71% low income (of three levels)	Primary care and community venues	Organisational:<br>- Collaborative care	Enhanced usual care (training update for clinicians)	(D) 12 months<br>(FU) 3, 6, 12 months	High (1/5 domains)	PHQ-9	None	None
Vera 2010, Puerto Rico	Age 18+ years<br>Depression (PHQ9≥10 and HSCL20 mean item score ≥1)	n = 179<br>Mean age: 55.2 years<br>76% female<br>SEP: "Predominantly low-income setting".	Primary care	Organisational:<br>- Collaborative care	Usual care	(D) 6 months<br>(FU) 2, 4, 6 months	Some	SCL-D20*	None	None

	DM, CVD, COPD, stroke, asthma, hypothyroidism, arthritis	50% high school education								
ORGANISATIONAL INTERVENTIONS: POST-DISCHARGE										
Markle-Reid 2021, Canada	Age 65+ years<br>Depression (CES-D≥10)<br>2+ self-reported LTCs<br>n = 127	n = 127<br>Mean age: 77.0 years<br>63% female<br>SEP: 59% low income (of two levels)	Home-based	Organisational:<br>- Post-discharge care<br>(Home-visits)	Usual care	(D) 6 months<br>(FU) 6 months	Some	CES-D-10<br>GAD-7	VR-12*	(G) VR-12 PCS
Schrader 2005, Australia	Age 18-84 years<br>Depression (CES-D≥16)<br>CHD, HF, arrhythmia	n = 669<br>Mean age: NR<br>38% female<br>SEP: NR	Primary care	Organisational:<br>- Post-discharge care<br>(Inter-professional case conference)	Usual care	(D) not specified<br>(FU) 12 months	Some	CES-D-10*	None	None
ORGANISATIONAL: STEPPED CARE										
Pols 2017, Netherlands	Age 18+ years<br>Subthreshold depression (PHQ9≥6, excl. MDD)<br>DM, CHD	n = 236<br>Mean age: 67.5 years<br>45% female<br>SEP: 38% low education level (of two levels)	Primary care	Organisational:<br>- Stepped care	Usual care	(D) 12 months<br>(FU) 3, 6, 9, 12 months	Some	PHQ-9[=]<br>HADS-D<br>HADS-A	None	None
Stoop 2015, Netherlands	Age 18+ years<br>Depression or anxiety (PHQ9≥7 or GAD7≥8)<br>DM, COPD, asthma	n = 46<br>Mean age: 59.5 years<br>50% female<br>SEP: 51% low education level (of two levels)	Primary care	Organisational:<br>- Stepped care	Usual care	(D) 12 months<br>(FU) 12, 18 months	Some	PHQ-9<br>GAD-7	None	None
PATIENT-ORIENTED INTERVENTIONS: EXERCISE										
Lamberti 2022, Italy	Age 40-75 years<br>Depression (MDD), anxious-depressive disorder (diagnosed)<br>“Chronic non-transmissible conditions”	n = 43<br>Mean age: 59.0 years<br>51% female<br>SEP: NR	Outpatient clinic (mental health)	Patient-oriented:<br>- Exercise<br>(Personalised exercise plan)	Usual care	(D) 10 months<br>(FU) 10 months	Some	None	SF-12*	(G) SF-12-PCS<br>(P) HbA1c[+], BMI, lipids

Tsang 2006, Hong Kong	Age 65+ years<br>Depression (diagnosis or raised GDS)<br>"Chronic medical illness"	n = 97<br>Mean age: 80.1 years<br>81% female<br>SEP: 67% illiterate	Community venues	Patient-oriented:<br>- Exercise<br>(Qigong traditional Chinese dance)	Attention control (newspaper reading group)	(D) 16 weeks<br>(FU) 2, 4, 5, 6 months	High (1/5 domains)	GDS	SQCPD personal wellbeing subscale	(G) SQCPD physical wellbeing subscale
Tsang 2013, Hong Kong	Age 65+ years<br>Depression (GDS≥8 or MDD)<br>"Chronic medical illness"	n = 38<br>Mean age: 60.3 years<br>68% female<br>SEP: 39% illiterate	Community venues	Patient-oriented:<br>- Exercise<br>(Qigong traditional Chinese dance)	Attention control (newspaper reading group)	(D) 12 weeks<br>(FU) 1-2, 3, 4, 5 months	Some	GDS<br>HDRS	SQCPD personal wellbeing subscale	(G) SQCPD physical wellbeing subscale
PATIENT-ORIENTED INTERVENTIONS: PSYCHOEDUCATION										
Ell 2017, USA	Age 18+ years<br>Depression (PHQ9≥10)<br>DM, CHD, HF	n = 348<br>Mean age: 56.5 years<br>85% female<br>SEP: 62% less than 6 years of schooling	Primary care and community venues	Patient-oriented:<br>- Psychoeducation	Enhanced usual care (written resources)	(D) 18 weeks<br>(FU) 6, 12 months	Some	PHQ-9<br>SCL-D20	None	(G) SF-12-PCS[+], Self-rating health<br>(P) HbA1C
Lamers 2010, Netherlands	Age 60+ years<br>Depression or dysthymia (MINI)<br>DM, COPD	n = 361<br>Mean age: 70.7 years<br>46% female<br>SEP: 35% low education (of two levels)	Home-based	Patient-oriented:<br>- Psychoeducation<br>(Home visits)	Usual care	(D) 12 weeks<br>(FU) 3, 6, 12 months	Some	BDI<br>SCL-90 (A)	SF-36[*]	(G) SF-36-PCS
McCusker 2016, Canada	Age 40+ years<br>Depression (PHQ9≥5)<br>1+ self-reported LTC, or chronic pain	n = 223<br>Mean age: 55.6 years<br>84% female<br>SEP: 34% low income (of two levels)	Online, telephone	Patient-oriented:<br>- Psychoeducation<br>(Self-help coaching)	Attention (access to self-care toolkit)	(D) 6 months<br>(FU) 3, 6 months	Some	PHQ-9	None	None
Raya-Tena 2021, Spain	Age 50+ years<br>Depression (BDI≥12)<br>DM, CHD, COPD, asthma	n = 380<br>Mean age: 68.4 years<br>82% female<br>SEP: 52% high deprivation (of two levels)	Primary care	Patient-oriented:<br>- Psychoeducation<br>(Group-based)	Usual care	(D) 12 weeks<br>(FU) 4, 12 months	Some	BDI	EQ-VAS	None
PATIENT-ORIENTED INTERVENTIONS: PSYCHOTHERAPY										
Araya 2021 Brazil & Peru	Age 21+ years<br>Depression (PHQ9≥10)<br>HTN, DM	n =<br>880 years (Brazil),<br>432 years (Peru)<br>Mean age:	App	Patient-oriented:<br>- Psychotherapy<br>(Digital, behavioural activation)	Usual care	(D) 6 weeks<br>(FU) 3, 6 months	Some	PHQ-8*	EQ-5D-3L	(F) WHODAS

		56.0 years (Brazil),<br>59.7 years (Peru)<br>% female:<br>87% (Brazil)<br>82% (Peru)<br>SEP: % low income (of two levels)<br>69% (Brazil)<br>72% (Peru)								
Cooper 2024, USA	Age 18+ years<br>Depression (PHQ9 ≥10)<br>HTN, DM, obesity	n = 69<br>Mean age: 38.1 years<br>80% female<br>SEP: 55% high deprivation (of four levels)	Primary care	Patient-oriented:<br>- Psychotherapy (Solution-focussed)	Attention control (equal contact)	(D) 3 weeks<br>(FU) 3 weeks	Some	PHQ-9<br>GAD-7	Flourishing Index	(P) HbA1c*, SBP⁺, BMI,
Johansson 2019, Sweden	Age 18+ years<br>Depression (PHQ9≥5)<br>AF, CHD, HF<br>n = 144<br>SES not reported	n = 144<br>Mean age: 63.0 years<br>62% female<br>SEP: 49% university education	Online	Patient-oriented:<br>- Psychotherapy (Digital, CBT)	Attention control (access to discussion forum)	(D) 9 weeks<br>(FU) 9 weeks	Some	PHQ-9*<br>MADR-S<br>GAD-7	EQ-VAS	None
Cully 2017, USA	Age: "Veterans"<br>Depression or anxiety (PHQ9≥10 or BAI≥16)<br>DM, CHD, HF	n = 302<br>Mean age: 65.5 years<br>6% female<br>SEP: 35% low income (of three levels)	Primary care	Patient-oriented:<br>- Psychotherapy (CBT)	Usual care	(D) 4 months<br>(FU) 4, 8 12 months	Some	PHQ-9<br>BAI	None	None
Monreal-Bartolome 2025, Spain	Age 18+ years<br>Depression (DSM-IV, excl. severe)<br>DM, chronic LBP	n = 183<br>Mean age: 51.4 years<br>72% female<br>SEP: 22-27% high deprivation (of four levels)	Primary care and online	Patient-oriented:<br>- Psychotherapy (Part-digital, mixed techniques)	Enhanced usual care (training update for clinicians)	(D) 8-12 weeks<br>(FU) 3, 6 months	Some	PHQ-9	SF-12	(F) RMD-Q<br>(P) HBA1C
Picariello 2024, UK	Age 18+ years<br>Depression (PHQ4≥3)<br>1+ self-reported LTC	n = 194<br>Mean age: 51.0 years<br>78% female<br>SEP: Median IMD decile 6 (IQR 4-9)	Online, telephone	Patient-oriented:<br>- Psychotherapy (Digital, CBT)	Usual care	(D) 12 weeks<br>(FU) 6, 12 weeks	Some	PHQ-9<br>GAD-7	EQ-5D-5L	(G) PGI-S<br>(F) WSAS
Pumar 2019, Australia	Age 18+ years<br>Depression or anxiety (GDS≥4 or GAI≥3)<br>ILD, COPD, asthma bronchiectasis	n = 65<br>Mean age: 68.8 years<br>60% female<br>SEP: NR	Rehabilitation service	Patient-oriented:<br>- Psychotherapy (Rehabilitation context, CBT)	Attention control (usual rehab, additional contact)	(D) 8-12 weeks<br>(FU) 9, 15 months	High (1/5)	GDS*<br>GAI*	None	(F) 6-min walk distance*

Ruesch 2017, Germany	Age 18+ years<br>Depressive episode, adjustment disorder with depression/anxiety, dysthymia<br>1+ self-reported LTC	n = 76<br>Mean age: 54.5 years<br>70% female<br>SEP: 4% did not finish education	Outpatient clinic (psychology)	Patient-oriented:<br>- Psychotherapy (Group-based, CBT)	Usual care	(D) 8 weeks<br>(FU) 2, 4 months	Some	HADS-D	SF-12*	SF-12 PCS
Sengupta 2024, India	Age 18-55 years<br>Depression/anxiety (DASS-D or DASS-A 'above cut off')<br>Eczema, psoriasis, acne, rosacea, vitiligo	n = 208<br>Mean age: NR<br>67% female<br>SEP: 0% high deprivation (of three levels)	Online	Patient-oriented:<br>- Psychotherapy (Digital, group-based, mindful self-compassion)	Usual care	(D) 4 weeks<br>(FU) 4 weeks	High (3/5 domains)	DASS-D<br>DASS-A	WHO Wellbeing Index	None
Wells 2021, UK	Age 18+ years<br>Depression or anxiety (HADS-A or -D ≥8)<br>CHD, HF, post-ICD, post-valve surgery, congenital heart disease	n = 332<br>Mean age: 60.3 years<br>35% female<br>SEP: 38% degree level education	Rehabilitation service	Patient-oriented:<br>- Psychotherapy (Rehabilitation context, group-based, meta-cognitive)	Usual rehab care	(D) 6 weeks<br>(FU) 4, 12 months	Some	HADS-D<br>HADS-A	EQ-5D-5L+<br>EQ-VAS	None
Wells 2023, UK	Age 18+ years<br>Depression or anxiety<br>CHD, HF, post-ICD, post-valve surgery, congenital heart disease	n = 240<br>Mean age: 60.6 years<br>40% female<br>SEP: 24% degree level education	Home-based	Patient-oriented:<br>- Psychotherapy (Rehabilitation context, self-help manual, meta-cognitive therapy)	Usual rehab care	(D) 4 months<br>(FU) 4 months	Some	HADS-D<br>HADS-A	EQ-5D-5L	None
Wilson 2018, USA	Age 18+ years<br>Depression (PHQ9≥10)<br>"Any chronic disease"	n = 47<br>Mean age: 46.7<br>85% female<br>SEP: 64% college degree	Online	Patient-oriented:<br>- Psychotherapy (Digital, mixed techniques)	Attention control (weekly contact)	(D) 8 weeks<br>(FU) 4, 8 weeks	Some	PHQ-9	None	(G) Self-rating health*

Table S4 Footnote

Studies ordered by intervention category (two groups), then intervention subtype, then alphabetically by author. Colour of study title column corresponds with components matrix (Figure 2 in main text).

Mental health outcomes include continuous measures of anxiety or depressive symptoms only (excludes distress, composite measures, mental component scores of wellbeing measures). Quality of life outcomes include generic measures only (exclude disease-specific measures). Physical health outcomes are grouped into global, physiological and functional outcomes, with physiological outcomes limited to the four most common (HbA1c, SBP, lipids, BMI)

[+] Indicates which outcome was used in the synthesis, when more than one outcome reported within the same category

* Indicates where data was not suitable for synthesis (e.g., depression outcome reported as binary; QOL reported as separate component scores; physical health outcome p-value unavailable)

Abbreviations (conditions): AF = atrial fibrillation, CHD = coronary heart disease, COPD = chronic obstructive pulmonary disease, CVD = cardiovascular disease, DM = diabetes mellitus, HF = heart failure, HTN = hypertension, ICD = implantable cardiac defibrillator, ILD = interstitial lung disease, LBP = low back pain, LTC = long term condition, MDD = major depressive disorder.

Abbreviations (outcome measures): see Supplementary Tables S1-S3

Other abbreviations: DSM-IV = diagnostic and statistical manual version 4, SEP = socioeconomic position (reported as economic status if available, otherwise education level), NR = not reported, CBT = cognitive behavioural therapy, IMD = index of multiple deprivation, MINI = mini international neuropsychiatric interview.

Figure S1. Risk of bias assessment

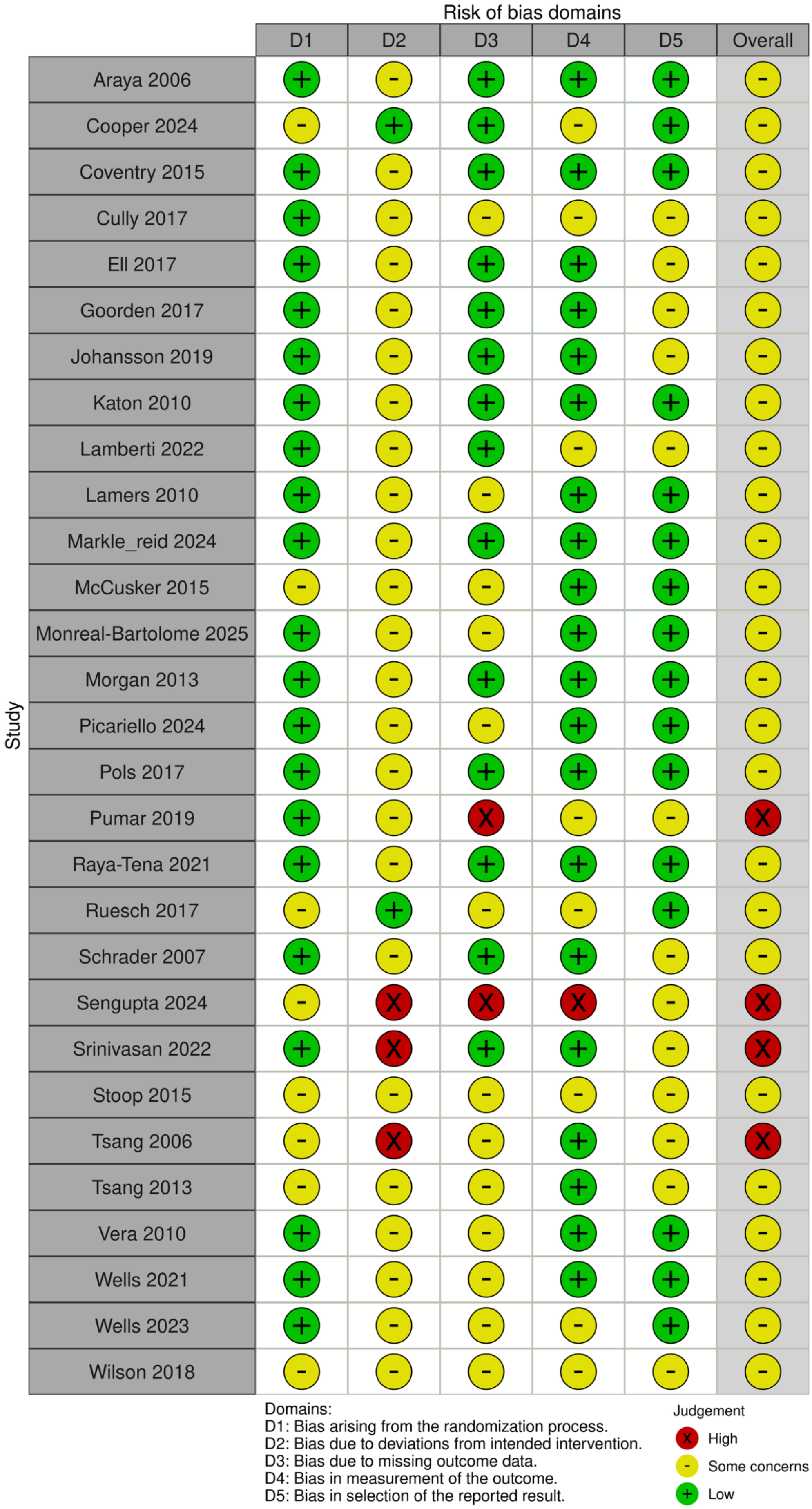

Figure S2. Funnel plots for synthesis groups with ten or more studies

(A) Effect of patient-level interventions on depression at end-intervention, including high risk of bias studies

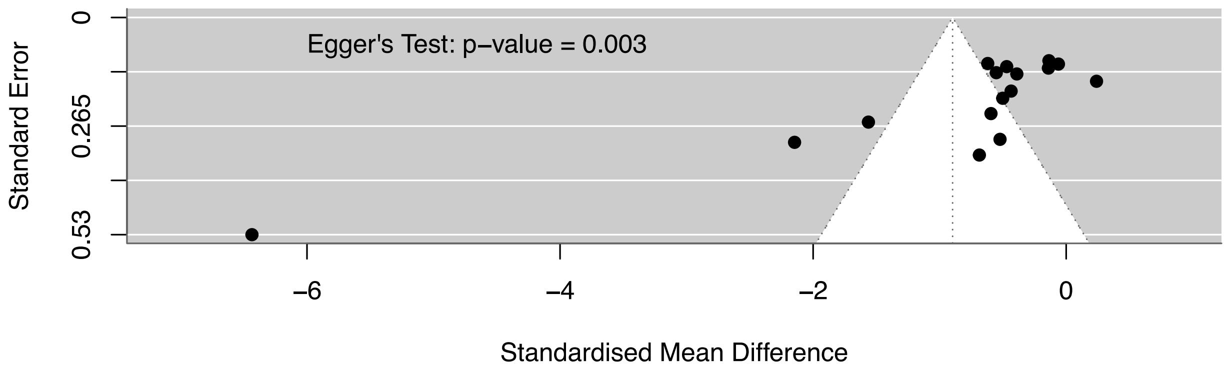

(B) Effect of patient-level interventions on depression at end-intervention, **excluding** high risk of bias studies

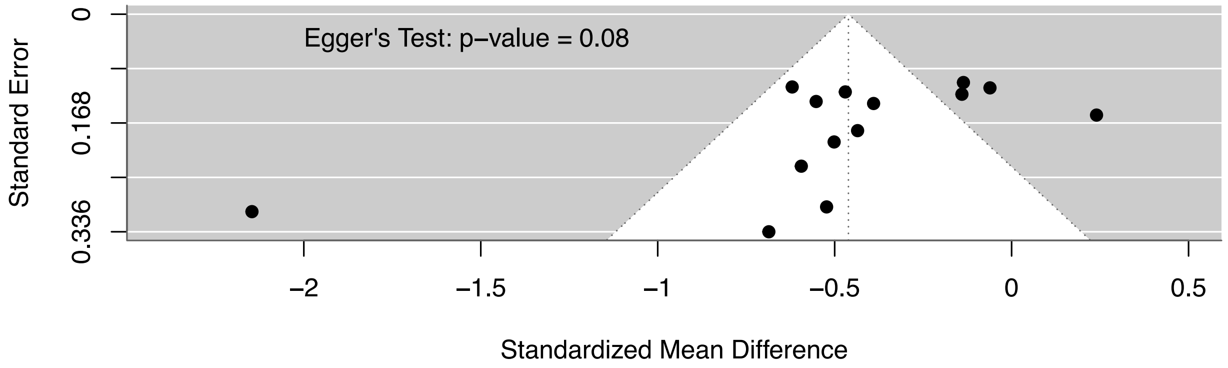

(C) Effect of patient-level interventions on quality of life at end-intervention, including high risk of bias studies

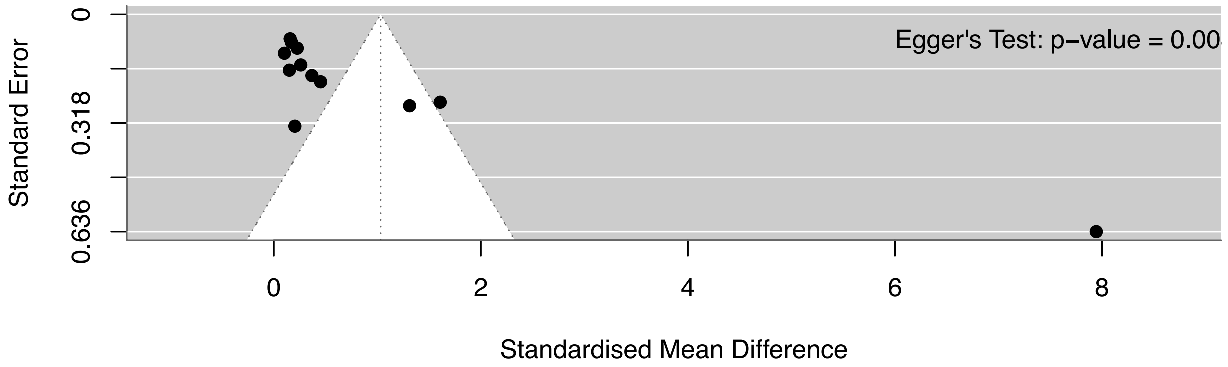

(D) Effect of patient-level interventions on quality of life at end-intervention, **excluding** high risk of bias

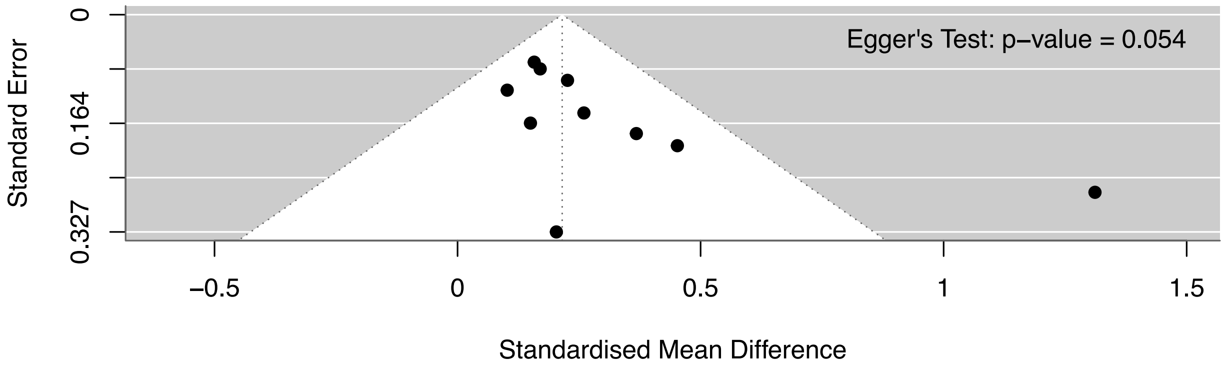

Table S5. Summary of findings of meta-analysis for all studies including high risk of bias.

Outcome	Assessment timing (range within group)	Effect of intervention on outcome (95% CI)	I^2 (%)	No. of participants (studies)	Quality of evidence (GRADE)	Effect size	Summary
ORGANISATIONAL INTERVENTIONS							
Depressive symptoms	End of intervention (4-12 months)	SMD 0.28 SD lower (0.43 lower to 0.13 lower)	59.32	3896 (7 RCTs)	Moderate a	Small	Organisational interventions **probably** result in a s**light reduction in depression at end-intervention.**
	Late follow up (18-24 months)	SMD 0.23 SD lower (0.53 lower to 0.08 higher)	68.85	883 (4 RCTs)	High	No significant effect	Organisational interventions result in **little to no difference in depression at late follow up.**
Anxiety symptoms	End of intervention (4-12 months)	SMD 0.11 SD lower (0.37 lower to 0.14 higher)	58.97	796 (4 RCTs)	High	No significant effect	Organisational interventions result in **little to no difference in anxiety at end-intervention.**
	Late follow up (18-24 months)	SMD 0.32 SD lower (1.23 lower to 0.59 higher)	84.58	282 (2 RCTs)	Low b,c	No significant effect	Organisational interventions **may** result in **little to no difference in anxiety at late follow up.**
Quality of life	End of intervention (4-12 months)	SMD 0.21 SD higher (0.01 higher to 0.41 higher)	21.15	682 (3 RCTs)	High	Small	Organisational interventions result in a **small improvement in quality of life at end-intervention.**
	Late follow up (18-24 months)	No studies reported data	-	-	-	-	No studies reported data
PATIENT-ORIENTED INTERVENTIONS							
Depressive symptoms	End of intervention (3wk – 6 months)	SMD 0.90 SD lower (1.61 lower to 0.19 lower)	99.56	3227 (16 RCTs)	Very low a,b,c,d,e	Large	These studies provide very uncertain evidence as to the effect of patient orientated interventions on depression at end-intervention.
	Late follow up (12 months)	SMD 0.17 SD lower (0.34 lower to 0.00 lower)	62.11	1723 (5 RCTs)	Moderate d	Trivial	Patient-orientated interventions result in a **trivial reduction in depression at late follow up.**
Anxiety symptoms	End of intervention (3wk – 4 months)	SMD 1.19 SD lower (2.40 lower to 0.02 higher)	93.38	1850 (8 RCTs)	Very low a,b,c,d	No significant effect	These studies provide very uncertain evidence as to the effect of patient orientated interventions on anxiety at end-intervention.
	Late follow up (12 months)	SMD 0.34 SD lower (0.48 lower to 0.21 lower)	0.00	995 (3 RCTs)	Moderate d	Small	Patient-orientated interventions result in a **slight reduction in anxiety at late follow up.**
Quality of life	End of intervention (3wk – 4 months)	SMD 1.03 SD higher (0.14 lower to 2.21 higher)	91.18	2897 (11 RCTs)	Very low a,b,c,d,e	Large	These studies provide very uncertain evidence as to the effect of patient orientated interventions on anxiety at end-intervention.
	Late follow up (12 months)	SMD 0.15 SD higher (0.08 lower to 0.39 higher)	54.90	856 (3 RCTs)	Moderate d	No significant effect	Patient-orientated interventions probably result in **little to no difference in quality of life at late follow up.**

Downgrading of evidence quality: (a) overall high risk of bias; (b) substantial inconsistency ($I^2 \geq 75\%$) in the results; (c) imprecision due to wide confidence interval (≥0.50 SD) (d) indirectness in study design (e.g., older adults only); (e) publication bias

Table S6. Summary of findings of subgroup meta-analysis (effects on intervention subtypes on depression, anxiety and quality of life, excluding high risk of bias studies)

Outcome	Timeframe	Effect of intervention on outcome SMD (95% CI)	I^2 (%)	No. of participants (studies)	Quality of evidence (GRADE)	Effect size	Summary
PATIENT-ORIENTED: EXERCISE							
Depressive symptoms	Short term (3-12 months)	-0.38 (-1.06 to 0.31)	n/a	38 (1 study)	Low b,c	No significant effect	Evidence suggests little to no difference
	Long term (18-24 months)	No data	-	-	-	-	No data in this timeframe
Anxiety symptoms	Short term (3-12 months)	No data	-	-	-	-	No data in this timeframe
	Long term (18-24 months)	No data	-	-	-	-	No data in this timeframe
Quality of life	Short term (3-12 months)	0.20 (-0.44 to 0.84)	n/a	38 (1 study)	Low b,c	No significant effect	Evidence suggests little to no difference
	Long term (18-24 months)	No data	-	-	-	-	No data in this timeframe
PATIENT-ORIENTED: PSYCHOEDUCATION							
Depressive symptoms	Short term (3-12 months)	0.00 (-0.20 to 0.19)	59.76	1312 (4 studies)	High	No significant effect	Results in little to no difference
	Long term (18-24 months)	No data	-	-	-	-	No data in this timeframe
Anxiety symptoms	Short term (3-12 months)	-0.04 (-0.25 to 0.16)	n/a	361 (1 study)	Moderate b	No significant effect	Likely results in little to no difference
	Long term (18-24 months)	No data	-	-	-	-	No data in this timeframe
Quality of life	Short term (3-12 months)	-0.01 (-0.24 to 0.21)	0.00	380 (1 study)	Moderate b	No significant effect	Likely results in little to no difference
	Long term (18-24 months)	No data	-	-	-	-	No data in this timeframe
PATIENT-ORIENTED: PSYCHOTHERAPY							
Depressive symptoms	Short term (3-12 months)	-0.38 (-0.55 to -0.21)	52.52	1327 (6 studies)	High	Small	Slight reduction in depression
	Long term (18-24 months)	No data	-	-	-	-	No data in this timeframe
Anxiety symptoms	Short term (3-12 months)	-0.67 (-1.11 to -0.23)	92.43	1281 (6 studies)	Moderate a	Medium	Likely reduces anxiety
	Long term (18-24 months)	No data	-	-	-	-	No data in this timeframe
Quality of life	Short term (3-12 months)	0.17 (0.09 to 0.25)	0.01	2405 (6 studies)	High	Trivial	Results in little to no difference
	Long term (18-24 months)	No data	-	-	-	-	No data in this timeframe
ORGANISATIONAL: COLLABORATIVE CARE							
Depressive symptoms	Short term (3-12 months)	-0.36 (-0.50 to -0.22)	0.00	1001 (3 studies)	High	Small	Slight reduction in depression
	Long term (18-24 months)	-0.30 (-0.51 to -0.09)	0.00	601 (2 studies)	High	Small	Slight reduction in depression
Anxiety symptoms	Short term (3-12 months)	-0.28 (-0.47 to -0.09)	n/a	387 (1 study)	High	Small	Slight reduction in anxiety
	Long term (18-24 months)	No data	-	-	-	-	No data in this timeframe
Quality of life	Short term (3-12 months)	0.22 (0.03 to 0.42)	17.50	682 (3 studies)	High	Small	Slight improvement in quality of life
	Long term (18-24 months)	No data	-	-	-	-	No data in this timeframe

ORGANISATIONAL: POST DISCHARGE							
Depressive symptoms	Short term (3-12 months)	0.03 (-0.37 to 0.42)	n/a	127 (1 study)	Low b,c	No significant effect	Evidence suggests little to no difference
	Long term (18-24 months)	No data	-	-	-	-	No data in this timeframe
Anxiety symptoms	Short term (3-12 months)	0.23 (-0.16 to 0.62)	n/a	127 (1 study)	Low b,c	No significant effect	Evidence suggests little to no difference
	Long term (18-24 months)	No data	-	-	-	-	No data in this timeframe
Quality of life	Short term (3-12 months)	No data	-	-	-	-	No data in this timeframe
	Long term (18-24 months)	No data	-	-	-	-	No data in this timeframe
ORGANISATIONAL: STEPPED CARE							
Depressive symptoms	Short term (3-12 months)	-0.19 (-0.81 to 0.43)	69.02	282 (2 studies)	Moderate c	No significant effect	Likely results in little to no difference
	Long term (18-24 months)	0.23 (-1.08 to 0.61)	82.46	282 (2 studies)	Low a,c	No significant effect	Evidence suggests little to no difference
Anxiety symptoms	Short term (3-12 months)	-0.16 (-0.59 to 0.27)	43.32	282 (2 studies)	Moderate c	No significant effect	Likely results in little to no difference
	Long term (18-24 months)	-0.32 (-1.23 to 0.59)	84.58	282 (2 studies)	Low a,c	No significant effect	Evidence suggests little to no difference
Quality of life	Short term (3-12 months)	No data	-	-	-	-	No data in this timeframe
	Long term (18-24 months)	No data	-	-	-	-	No data in this timeframe

Excludes high risk of bias studies (n=4)

Downgrading of evidence quality: (a) substantial inconsistency (I2≥75%) in the results; (b) indirectness due to study design (e.g., only older adults); (c) imprecision due to wide confidence interval (≥0.50 SD)

Figure S3. Meta-analysis of effect of interventions on depressive symptoms at end-intervention and late follow-up.

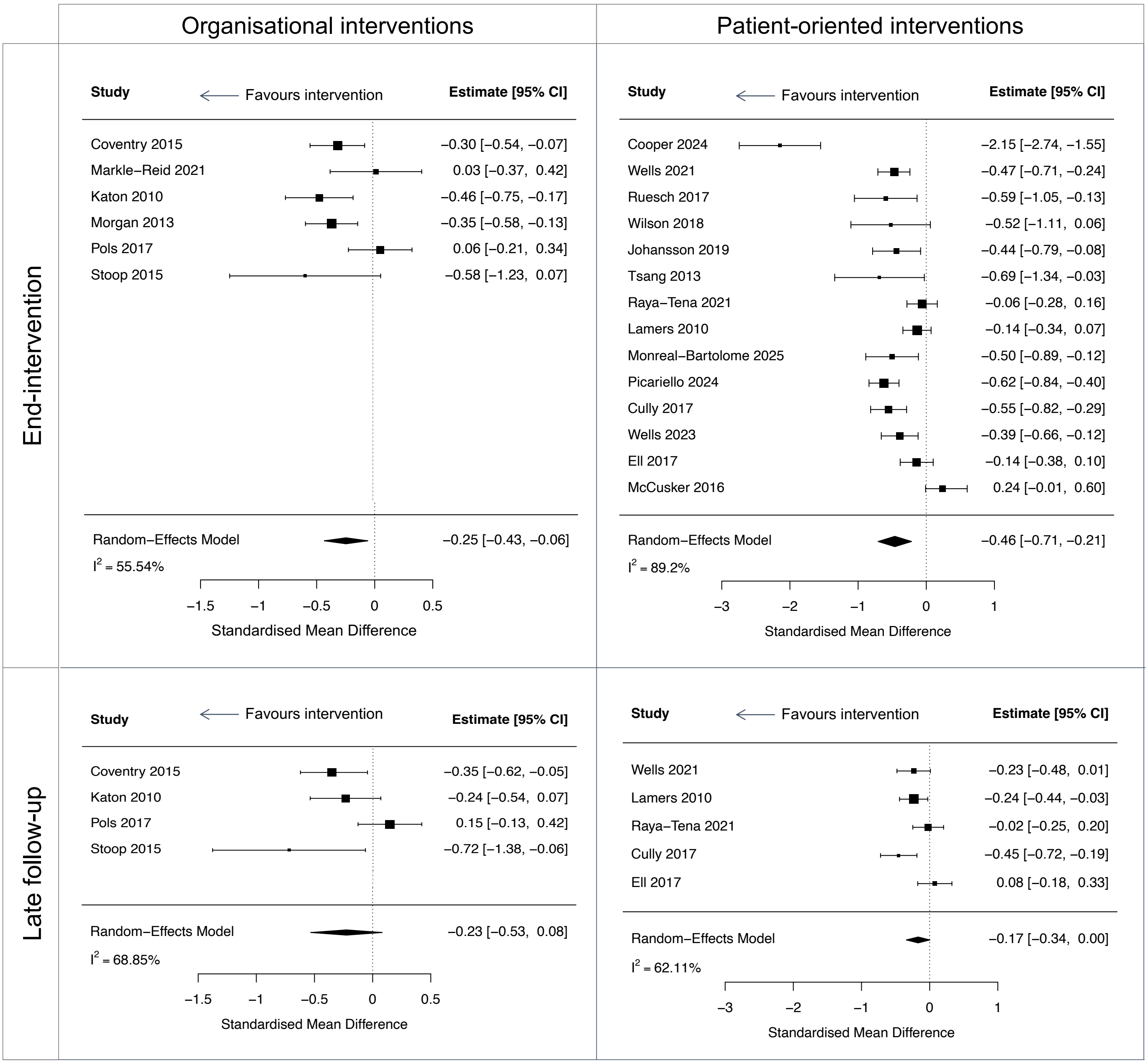

NB. Excludes high risk of bias studies

‘End-intervention’ refers to the closest assessment time point to the end of the intervention, which spanned 4-12 months for organisational interventions, and 3 weeks to 4 months for patient-oriented interventions.

Late-follow was 12 months for patient-oriented interventions, and 18-24 months for organisational interventions.

Figure S4. Meta-analysis of effect of intervention subtypes on quality of life at 3-12 months

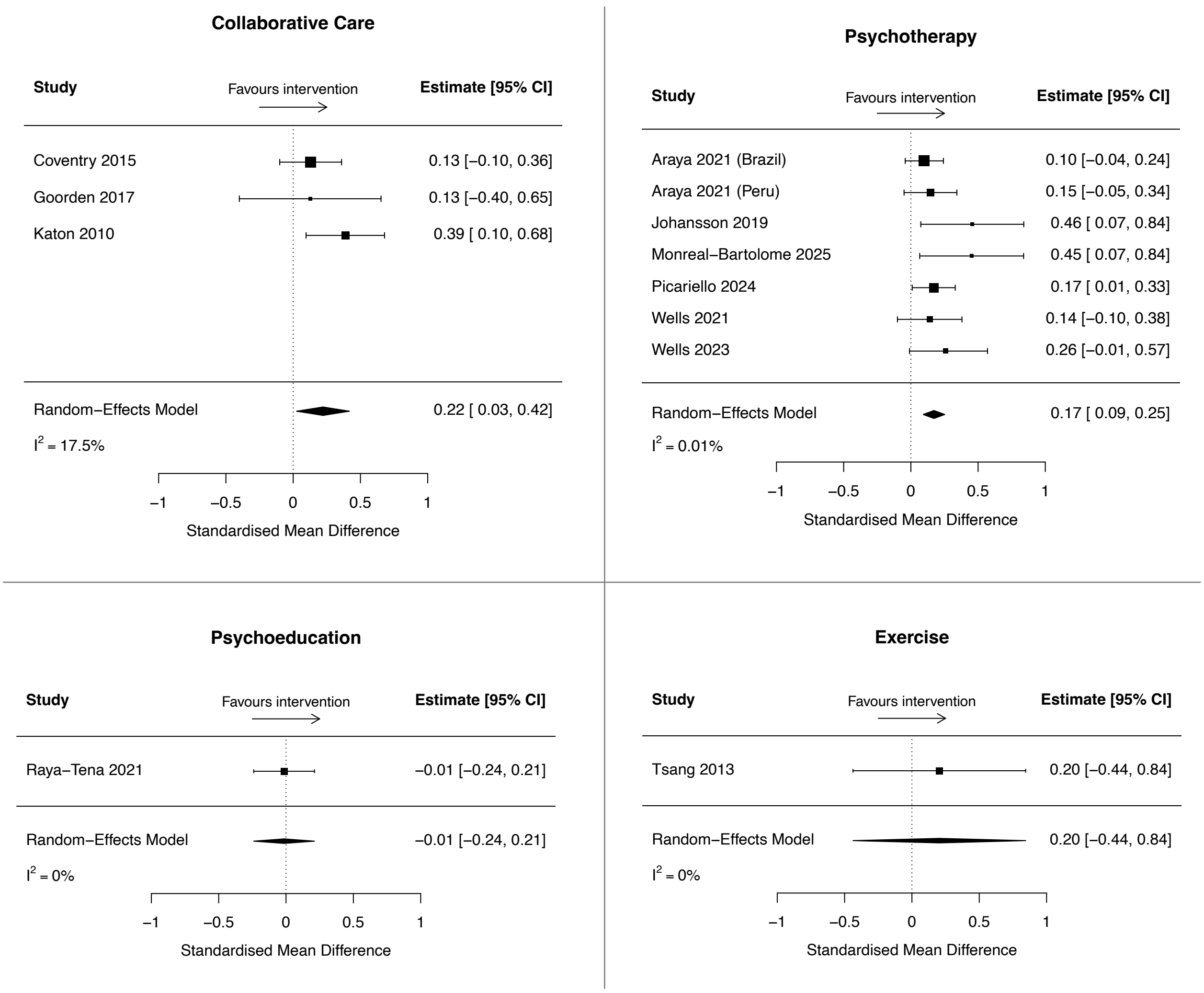

NB. Excludes high risk of bias studies

Figure S5. Effect of organisational interventions on anxiety at late follow-up (18-24m)

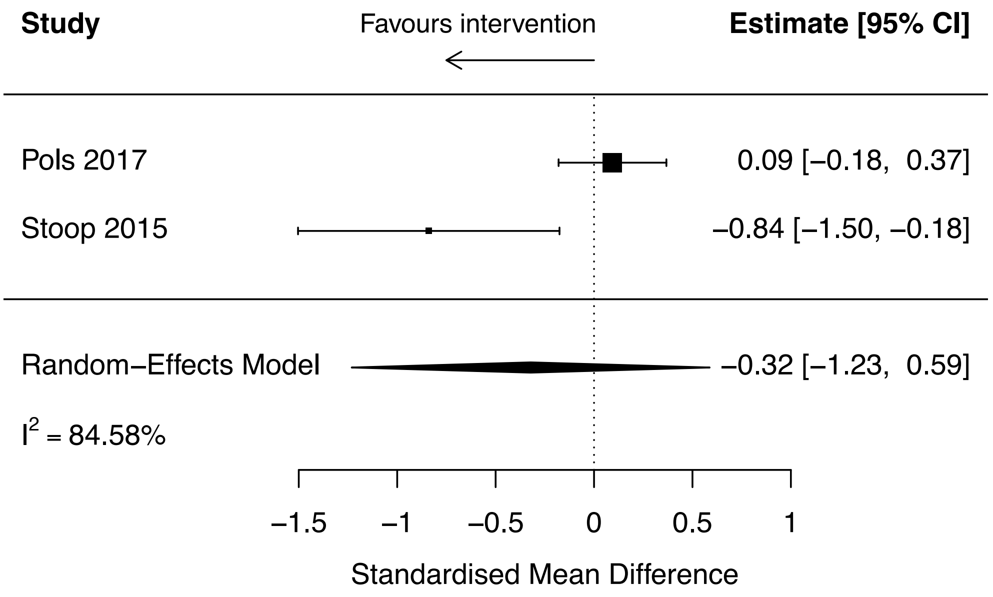

NB. No studies in this group were at high risk of bias

Figure S6. Effect of patient-level interventions on anxiety at end-intervention (3wk – 6m), including high risk of bias studies (red dot)

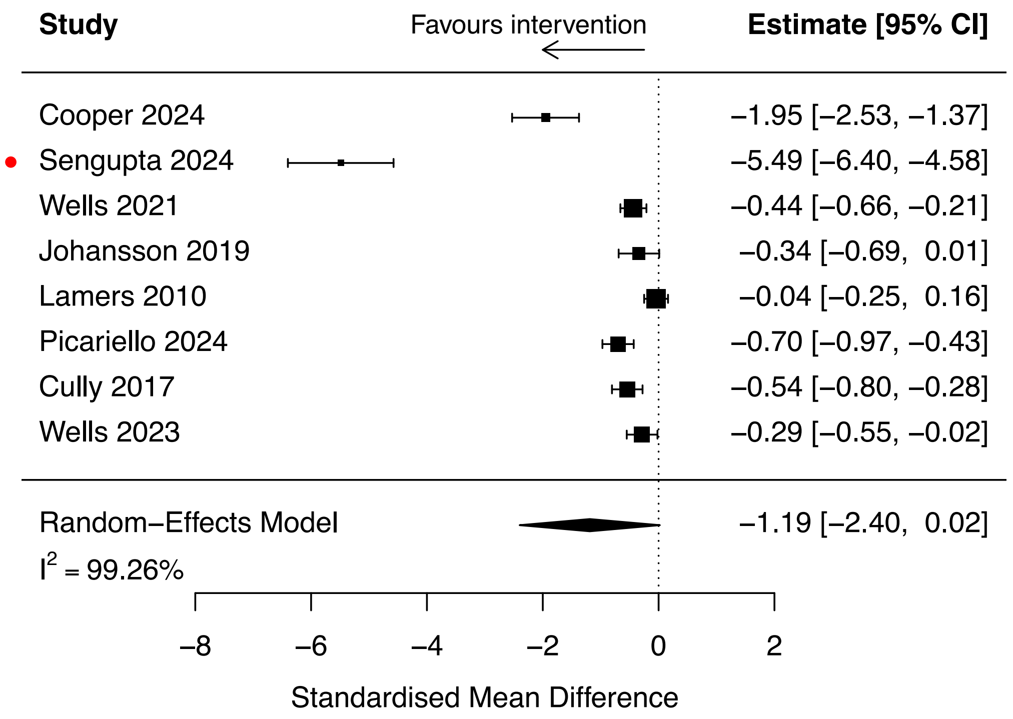

Figure S7. Effect of patient-level interventions on anxiety at late follow-up (12m)

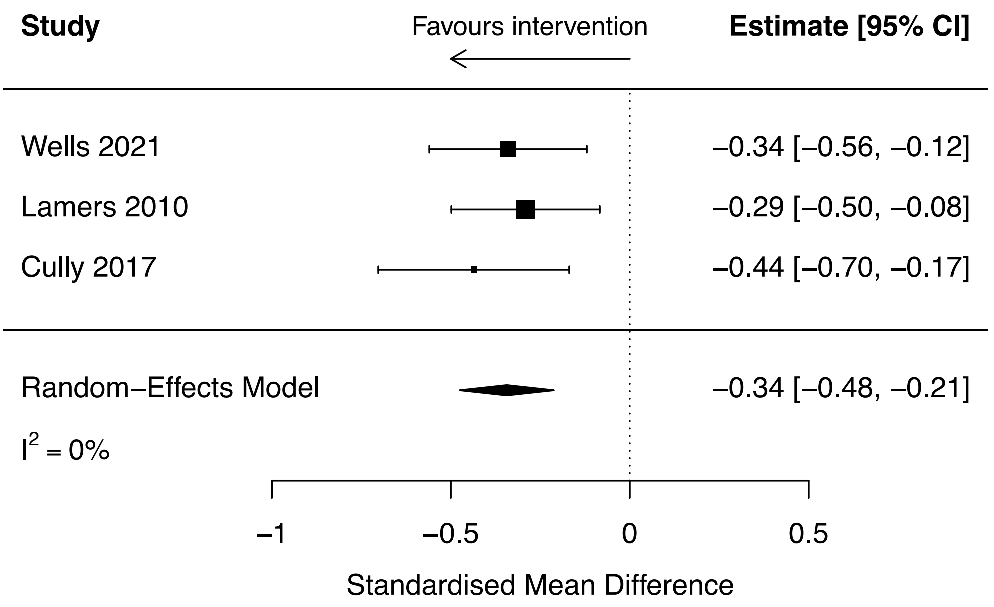

NB. No studies in this group were at high risk of bias

Figure S8. Effect of organisational interventions on depression at end-intervention (4-12m), including high risk of bias studies (red dot)

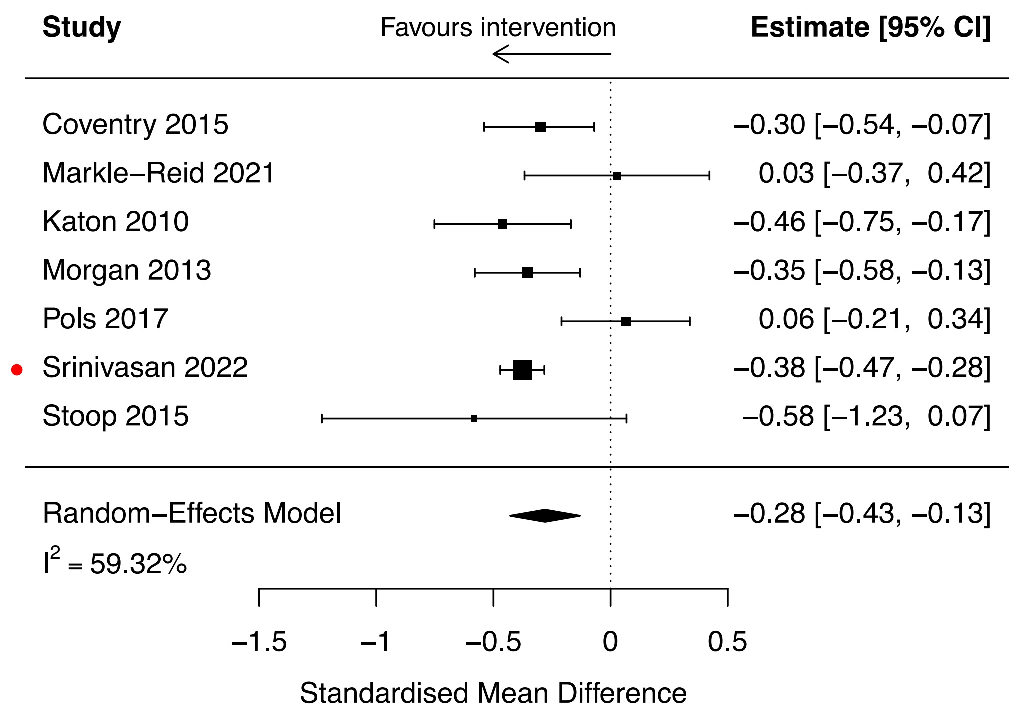

Figure S9. Effect of patient-level interventions on quality of life at end-intervention (3wk – 6m), including high risk of bias studies (red dot)

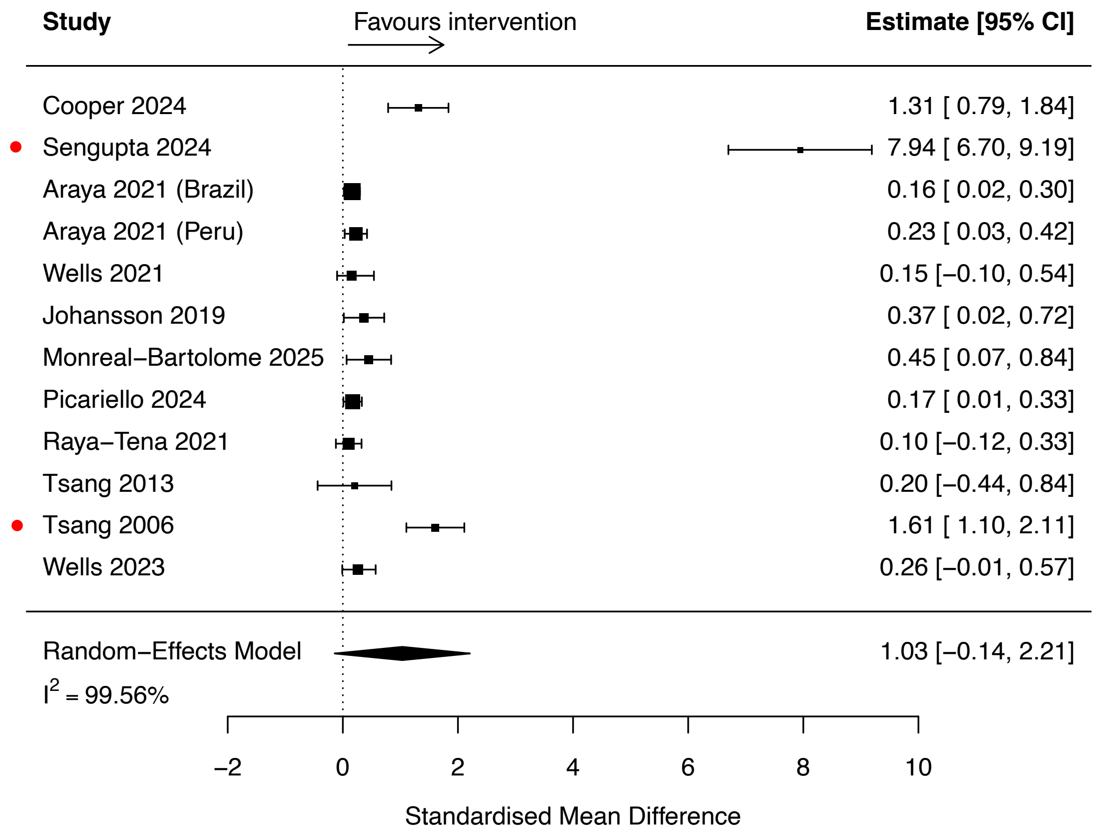

Figure S10. Effect of patient-level interventions on quality of life at late follow-up (12m)

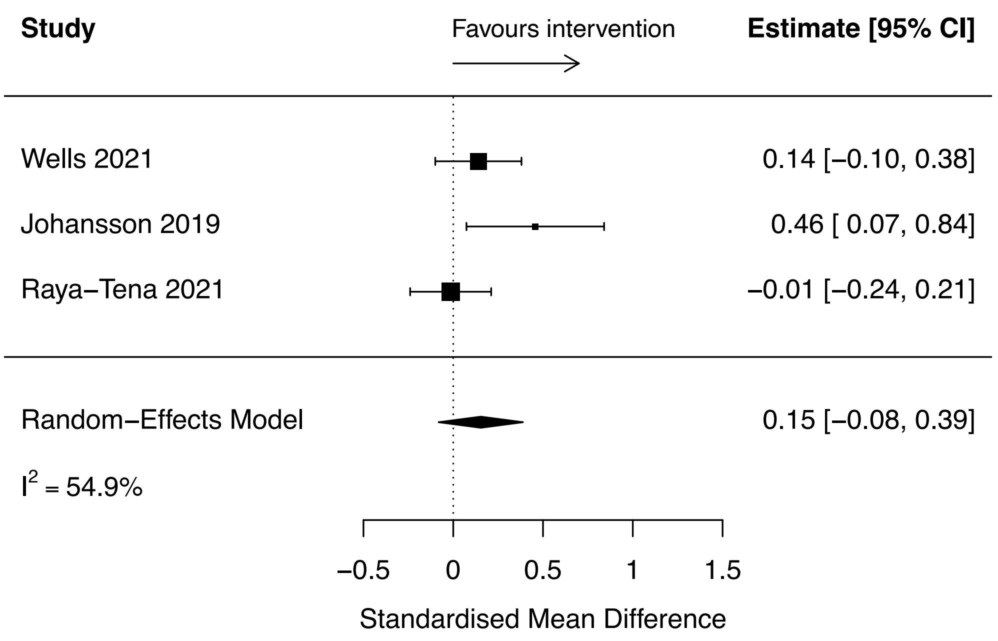

NB. No studies in this group were at high risk of bias

Figure S11. Effect of patient-level interventions on depression at end-intervention (3wk – 6m), including high risk of bias studies (red dot)

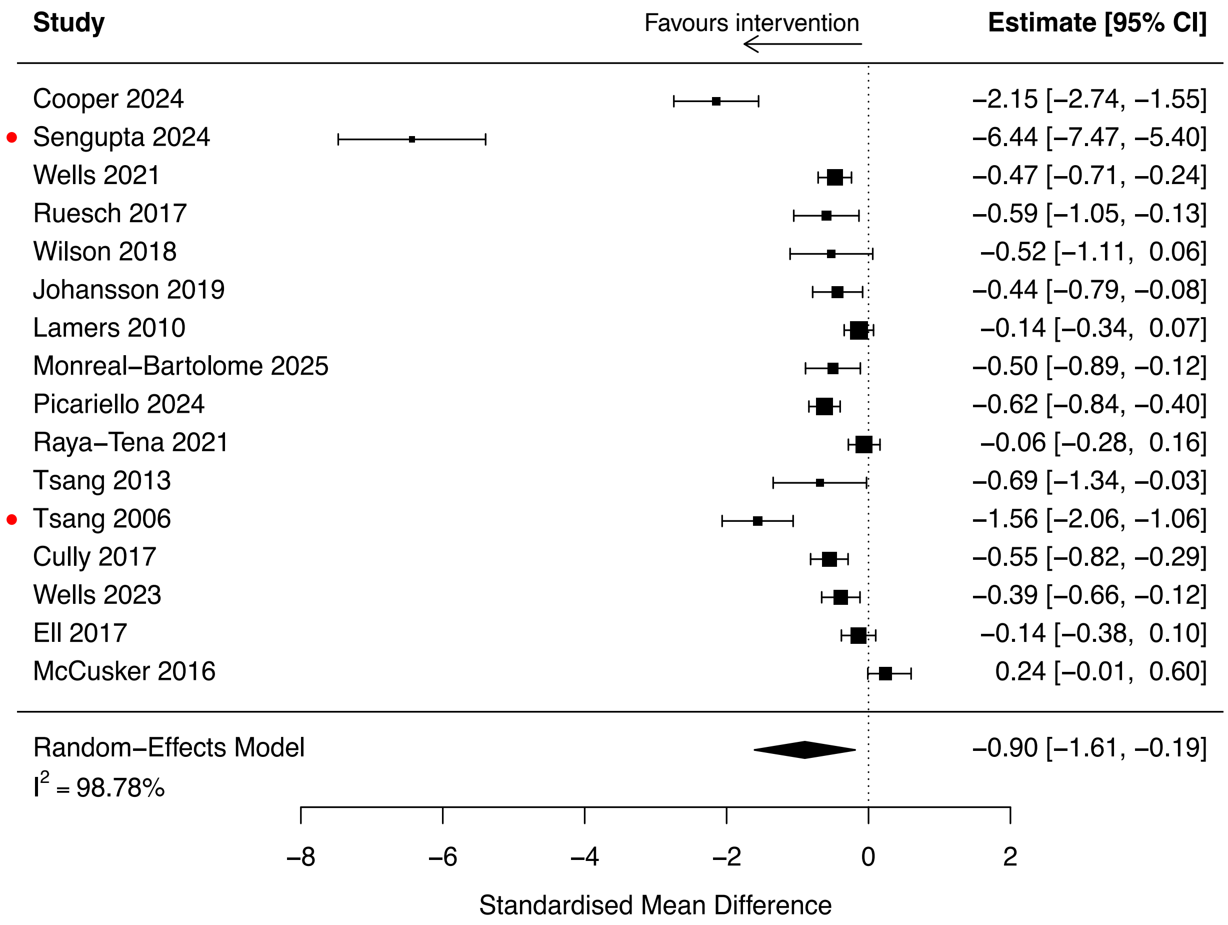

Table S7. Effects on physical health outcomes, grouped as global, functional and physiological.

Study	Outcome measure	Outcome type	Direction of effect	2-sided p-value	Sample size
Predominantly organisational interventions					
Coventry 2015	SDS	Functional	Favours intervention	0.24	316
Katon 2010	WHO-DAS-II	Functional	Favours intervention	0.2	184
Katon 2010	Physiological markers (composite)	Physiological	Favours intervention	0.001	210
Markle-Reid 2021	VR-12-PCS	Global	Favours control	0.42	99
Morgan 2013	HbA1C	Physiological	Favours intervention	0.049	156
Predominantly patient-oriented interventions					
Araya 2021 (Brazil)	WHODAS-II	Functional	Favours intervention	0.02	880
Araya 2021 (Peru)	WHODAS-II	Functional	Favours intervention	0.001	432
Cooper 2024	SBP	Physiological	Favours intervention	0.06	49
Ell 2017	SF-12-PCS	Global	Favours control	0.98	263
Ell 2017	HbA1c	Physiological	Favours intervention	0.52	314
Johansson 2019	SF-12-PCS	Global	Favours intervention	0.06	144
Lamberti 2022	SF-12-PCS	Global	Favours intervention	0.002	43
Lamberti 2022	HbA1c	Physiological	Favours intervention	0.051	43
Lamers 2010	SF-36-PCS	Global	Favours intervention	0.42	219
Monreal-Bartolome 2025	RMDQ	Functional	Favours intervention	0.43	77
Monreal-Bartolome 2025	HbA1c	Physiological	Favours control	0.6	21
Picariello 2024	WSAS	Functional	Favours intervention	0.01	152
Picariello 2024	PGI-S	Global	Favours control	0.438	151
Ruesch 2017	SF-12-PCS	Global	Favours intervention	0.001	76
Tsang 2006*	SQCPD (Physical wellbeing scale)	Global	Favours intervention	0.001	82
Tsang 2013	SQCPD (Physical wellbeing scale)	Global	Favours intervention	0.048	38

Where p-values reported as <0.001, the upper limit was used for synthesis (combining p-values)
Abbreviations of outcome measures: see Supplementary Table S3
*Tsang 2006 = high risk of bias

Table S8. Results of Fisher's method for combining p-values by intervention group, including high risk of bias studies

Type of physical health outcome	Combined p-value (number of studies)
Organisational interventions	
Functional	0.065 (two studies)
Global	One study
Physiological	<0.001 (two studies)
Patient-oriented interventions	
Functional	<0.001 (four studies)
Global*	<0.001 (eight studies)*
Physiological	0.023 (four studies)

*NB: This synthesis includes one result from a high risk of bias study, in the patient-oriented interventions, global outcomes category. When this result is excluded, the result of Fisher's synthesis remains the same (combined p <0.001, from seven studies)

Table S9. Results of Fisher's method for combining p-values by intervention subtype, including high risk of bias studies

	Type of physical health outcome		
Intervention subtype	Functional	Global	Physiological
Collaborative care	0.065 (two studies)	No data	<0.001 (two studies)
Stepped Care	No data	No data	No data
Post-discharge	No data	One study	No data
Exercise	No data	<0.001 (three studies)*	One study
Psychoeducation	No data	0.346 (two studies)	One study
Psychotherapy	<0.001 (four studies)	<0.001 (three studies)	0.102 (two studies)

*NB: This synthesis includes one result from a high risk of bias study, in the exercise interventions, global outcomes category. When this result is excluded, the result of Fisher's synthesis remains the same (combined p <0.001, from two studies)

Box S3. Reasons for exclusion from meta-analysis

- Araya 2021 and Schrader 2007 both reported results for depression in a dichotomous format (proportion achieving a specified reduction from baseline, or proportion with no vs mild vs moderate depression after treatment), with output including either odds ratio or relative risk.
- Lamberti 2022 reported SF-12 mental component and physical component scores separately. These could not simple be combined because the covariance, which is needed to calculate the standard deviation of the composite score, is not known.
- Pumar 2019 reported depression scores in the intervention and control groups as medians and interquartile ranges.
- Vera 2010 reported results in the form of mixed-effects regression estimates, with insufficient information to confidently know how the model was specified.

Table S10. Overview of studies in older populations with depression where the presence of a physical long-term conditions was not stipulated.

**Study**	**Population**	**Intervention**	**Comparator**	**Primary outcome(s)**	**Results summary**
Banerjee 1996[9]	n=69<br>65+ years<br>Symptoms of depression	Organisational (care coordination). MDT and key worker.	Usual care	Depression	Statistically significant improvement in depression at 6 months
Ciechanowski 2004[10]	n=138<br>60+ years<br>Symptoms of depression	Psychological therapy. Home-based problem-solving therapy.	Usual care	Depression and QoL	Statistically significant improvement in depression at QoL at 12 months
Chew Graham 2007 [11]	n=105<br>60+ years<br>Symptoms of depression	Collaborative care, including guided self-help	Usual care	Depression	No statistically significant effect on depression
Ell 2007[12]	n=311<br>65+ years<br>Symptoms of depression	Stepped care involving medication and psychological therapy.	Enhanced usual care	Depression and QoL	No statistically significant effect on depression or QoL
Bruce 2015[13]	n=306<br>65+ years<br>Symptoms of depression	Organisational (care coordination). Nurse home visits.	Enhanced usual care	Depression	No statistically significant effect on depression

References

1. Clark JM, Sanders S, Carter M, Honeyman D, Cleo G, Auld Y, et al. Improving the translation of search strategies using the Polyglot Search Translator: a randomized controlled trial. J Med Libr Assoc. 2020;108(2):195-207.
2. Smith SM, Wallace E, O'Dowd T, Fortin M. Interventions for improving outcomes in patients with multimorbidity in primary care and community settings. Cochrane Database of Systematic Reviews. 2016;2016(3).
3. Bricca A, Harris LK, Jäger M, Smith SM, Juhl CB, Skou ST. Benefits and harms of exercise therapy in people with multimorbidity: A systematic review and meta-analysis of randomised controlled trials. Ageing Res Rev. 2020;63:101166.
4. Lukens EP, McFarlane WR. Psychoeducation as evidence-based practice: Considerations for practice, research, and policy. Brief Treatment and Crisis Intervention. 2004;4(3):205-25.
5. Pawlak A, Kacprzyk-Straszak A. What is psychotherapy today? Overview of psychotherapeutic concept. Journal of Education, Health and Sport. 2020;10:19.
6. Archer J, Bower P, Gilbody S, Lovell K, Richards D, Gask L, et al. Collaborative care for depression and anxiety problems. Cochrane Database of Systematic Reviews. 2012.
7. Jeitani A, Fahey PP, Gascoigne M, Darnal A, Lim D. Effectiveness of stepped care for mental health disorders: An umbrella review of meta-analyses. Personalized Medicine in Psychiatry. 2024;47-48.
8. Marini G, Longhini J, Ambrosi E, Canzan F, Konradsen H, Kabir ZN. Transitional Care Interventions in Improving Patient and Caregiver Outcomes After Discharge: A Scoping Review. Healthcare (Basel). 2025;13(3).
9. Banerjee S, Shamash K, Macdonald AJ, Mann AH. Randomised controlled trial of effect of intervention by psychogeriatric team on depression in frail elderly people at home. Bmj. 1996;313(7064):1058-61.
10. Ciechanowski P, Wagner E, Schmaling K, Schwartz S, Williams B, Diehr P, et al. Community-Integrated Home-Based Depression Treatment in Older Adults: A Randomized Controlled Trial. JAMA. 2004;291(13).
11. Chew-Graham CA, Lovell K, Roberts C, Baldwin R, Morley M, Burns A, et al. A randomised controlled trial test the feasibility of a collaborative care model for the management of depression in older people. British Journal of General Practice. 2007;57(538).
12. Ell K, Unützer J, Aranda M, Gibbs NE, Lee PJ, Xie B. Managing depression in home health care: a randomized clinical trial. Home Health Care Serv Q. 2007;26(3):81-104.
13. Bruce ML, Raue PJ, Reilly CF, Greenberg RL, Meyers BS, Banerjee S, et al. Clinical Effectiveness of Integrating Depression Care Management Into Medicare Home Health: The Depression CAREPATH Randomized Trial. JAMA Internal Medicine. 2015;175(1):55-64.
