# Supplementary material for: Primary care and community interventions for multimorbidity involving depression or anxiety: systematic review with meta-analysis
Source: BMJ Med. 2026 Apr 10;5(1):e002400. doi: 10.1136/bmjmed-2025-002400 (PMC13084858; doi:10.1136/bmjmed-2025-002400)
Supplement: Supplementary file 4 [file bmjmed-2025-002400.reviewer_comments.pdf]

PEER REVIEW HISTORY

BMJ Medicine publishes all reviews undertaken for accepted manuscripts. Reviewers are asked to complete a checklist review form and are provided with free text boxes to elaborate on their assessment. These free text comments are reproduced below.

This paper was submitted to another journal from BMJ but declined for publication following peer review. The authors addressed the reviewers' comments and submitted the revised paper to BMJ Medicine. The paper was subsequently accepted for publication at BMJ Medicine.

ARTICLE DETAILS

**TITLE (PROVISIONAL)**	Primary care and community interventions for multimorbidity involving depression or anxiety: A systematic review with meta-analysis
**AUTHORS**	Sweeney, Kieran; Gilarova, Michaela; Ng, Lauren; Baker, Jennifer; Maxwell, Susanne; Macrae, Clare; Mercer, Stewart; Anand, Atul; Guthrie, Bruce; Stirland, Lucy

VERSION 1 - REVIEW

**REVIEWER NAME**	Bricca, Alessio
**REVIEWER AFFILIATION**	University of Southern Denmark
**REVIEWER CONFLICT OF INTEREST**	None
**DATE REVIEW RETURNED**	20-Oct-2025

**GENERAL COMMENTS**	Thank you for the opportunity to review this interesting and timely manuscript. My comments primarily focus on the methodology of the systematic review and the interpretation of findings from the exercise trials in patients with multimorbidity, which is my main area of expertise. Please find my detailed comments for the authors below.<br><br>Major Comments<br>1. Inclusion Criteria: While the rationale for selecting depression and anxiety as representative mental disorders is clear, it would be helpful if the authors could further clarify the reasoning behind including any physical condition as part of the criteria. The intro states that there is a close association between mental health disorders and many (but not all) physical conditions.<br><br>2. Rationale for Grouping: I commend the authors for providing detailed descriptions of the intervention components. While this is very useful for descriptive purposes, conducting a meta-analysis that combines all patient-oriented interventions and reporting a single SMD as primary results may be misleading. For example, although exercise, psychotherapy, and psychoeducation are all patient-directed interventions, they differ significantly in modality, underlying constructs, and rationale (as also noted by the authors in Box 2). This makes it challenging to analyse them together.<br><br>3. Exercise Trials: Three trials are categorised as exercise trials (Figure 2 and Table S4). Two of these (Tsang 2006 and Tsang 2013) involve Qigong (traditional Chinese dance), while one

	(Lamberti) features a more "conventional" exercise program. Only the Tsang studies are included in the meta-analysis, so it may be inappropriate to draw conclusions about exercise in general, as these studies focus on Qigong. I would also like to draw the authors' attention to a recently published trial on exercise for multimorbidity (MOBILIZE; https://www.nature.com/articles/s41591-025-03779-4), where I served as senior author. Please consider whether this study could enrich the introduction/discussion on exercise for this population and provide a useful comparison, despite differences in the exercise interventions included in this review and the MOBILIZE trial.<br><br>4. SMD Estimation: The Lamberti study is not included in the meta-analysis. Upon reviewing the paper, it appears that the necessary data are available in Table 2 and the bar graph for SF-12. Since SF-12 is considered comparable to other HRQoL measures (Table S1), could the authors clarify whether it was not possible to estimate the SMD for this study?<br><br>5. Also, is it feasible to estimate SMDs from other studies included in the Albatross plot (i.e., those excluded from the meta-analysis)? As SMDs can sometimes be estimated from p-values, graphs or other methods https://ma-accelerator.com/ should these studies be considered for inclusion in the meta-analyses?<br><br>6. Risk of bias. I checked again the Lamberti study because in the Risk of Bias D5 domain it was deemed as Low risk of bias by the authors but I could not find their protocol in clinicaltrials.gov or similar database or in the original paper. Should this domain be deemed as 'some concerns' or 'high risk of bias' instead?<br><br>7. GRADE Assessment: I would like the authors to clarify the rationale for not downgrading for Inconsistency and Indirectness in most meta-analyses. For example, in patient-oriented interventions, the certainty of evidence is rated as moderate to high for depression. Given the limited number of trials, differences in interventions and the fact that inconsistency could not be explained by removing high-risk-of-bias studies or further investigation for example with meta-regression analyses, this rating may be overly optimistic. Also, is the population included in the RCTs representative of the broader target population (i.e., individuals with multimorbidity: depression/anxiety and at least one chronic condition)? If not, then one could consider downgrading also for Indirectness.<br><br>8. Change Scores Clarification: Could the authors please clarify whether the reported effects are based on end-of-treatment scores, change scores, or both?<br><br>Minor Comments<br><br>1. Consider briefly reporting the certainty of evidence in the Results section, rather than only in the Discussion (e.g., see the first paragraph of the Discussion).<br><br>2. PPI: It is commendable that patients were involved in the systematic review. However, more detail on the number, characteristics, and representativeness of included participants would help readers understand and potentially reproduce this aspect of the methodology.

	3. PRISMA Compliance: Please consider explicitly stating that the review follows the PRISMA checklist.<br><br>4. Comparator: Consider briefly describing the characteristics of the comparators in the Results section to better contextualise the intervention effects.<br><br>5. Meta-Regression Analyses: Please mention that the heterogeneity observed in the meta-analysis could not be further explored, for example, via meta-regression analyses.

**REVIEWER NAME**	Calderón-Larrañaga, Amaia
**REVIEWER AFFILIATION**	Aging Research Center, Karolinska Institutet
**REVIEWER CONFLICT OF INTEREST**	None
**DATE REVIEW RETURNED**	20-Oct-2025

**GENERAL COMMENTS**	This is a very interesting and carefully conducted study, focusing on a group of patients with physical–mental multimorbidity who represent some of the greatest complexity and risk of adverse outcomes in today's healthcare systems. The topic is highly relevant and timely, and I commend the authors for their rigorous work. Below, I provide several suggestions that may help strengthen the manuscript further:<br>• I recommend making it clear in the title that the focus is on primary care and/or community-based interventions, to better reflect the scope and narrow down the focus of the review.<br>• The introduction notes that individuals with both physical and mental health conditions experience poorer quality of life and physical health outcomes, which justifies interventions targeting this group. However, it remains somewhat unclear how outcomes for people with both physical and mental health conditions differ from those with (i) only mental health conditions, or (ii) one mental and one physical condition. Strengthening the rationale for specifically targeting persons with combined physical–mental multimorbidity would make the case more compelling.<br>• Measures of distress and mental components of well-being were not included, while several measures of physical well-being and self-reported health were considered. An explanation for this asymmetry would be valuable. More broadly, the justification of outcome selection could be elaborated. Interventions of this type often include outcomes linked to psychosocial well-being, patient health behaviors, healthcare utilization, etc. While I understand the chosen end points, a stronger rationale for their prioritization would improve clarity.<br>• I recommend adding a table summarizing definitions of each intervention type and subtype, to help readers navigate the evidence. I was surprised that self-management interventions were not highlighted among the patient-oriented approaches.<br>• The exclusion of studies focused on older adults with mental health problems is concerning, given that this is often the group with the highest levels of complexity, due to multiple coexisting conditions, functional impairment, and unique manifestations of late-life mental health. Could the authors provide the number of studies excluded on this basis, and at least a brief narrative summary of their findings?<br>• The role of collaborative care could also be discussed more explicitly, as it is often central in managing complex multimorbidity,

	and happens to be one of intervention types holding biggest potential.<br>• The authors conclude that the evidence for organizational and patient-oriented interventions is limited, particularly in the long term. It would be valuable if the discussion could be extended to recommendations for future intervention design, for example: content (should future approaches combine organizational and patient-oriented strategies?); duration (is longer follow-up, e.g., ≥2 years, needed?); target groups (should studies explicitly focus on multimorbid populations with ≥2 conditions, including at least one mental health condition?); and design (given that lack of blinding was a common source of bias, are there methodological strategies to address this?).<br>• Finally, I recommend a careful editorial review to correct small typographical errors that appear throughout the text.

**REVIEWER NAME**	Reviewer 3
**REVIEWER AFFILIATION**	
**REVIEWER CONFLICT OF INTEREST**	None
**DATE REVIEW RETURNED**	20-Oct-2025

**GENERAL COMMENTS**	This SRMA aimed to identify effective interventions targeting individuals with multimorbidity, specifically those with depression/anxiety and comorbid physical conditions. The primary outcomes considered were improvements in quality of life and reductions in depressive and anxiety symptoms. The methodology is rigorous, with a well-conducted search strategy and clearly documented selection steps. The meta-analysis was performed to high standards, and the subgroup analyses are well justified.<br><br>The introduction highlights the UK context, yet the SRMA itself is not geographically restricted. It is unclear why the UK is singled out in framing the background. Does the prevalence or healthcare organization in the UK differ in a way that justifies this focus? Similarly, the discussion refers specifically to NICE recommendations. Could these points be framed within a broader, more international context?<br><br>To enhance clinical relevance, the discussion would benefit from deeper consideration of which comorbid diseases—or clusters of diseases—interact most strongly with specific interventions. This would help clarify the mechanisms through which interventions exert their effects in multimorbid populations.<br><br>In the section on knowledge gaps and future directions, the concept of multimorbidity patterns or clusters is notably absent. Recent literature increasingly supports this approach as a way to stratify populations and identify the most appropriate target groups for interventions. Focusing only on individual comorbidities may not capture the complexity of multimorbidity, and patterns-based approaches could provide more meaningful insights for tailoring interventions.

**REVIEWER NAME**	Reviewer 4
**REVIEWER AFFILIATION**	

**REVIEWER CONFLICT OF INTEREST**	None
**DATE REVIEW RETURNED**	20-Oct-2025

**GENERAL COMMENTS**	Summary:<br>I want to thank the authors for the opportunity to review their study on identifying and characterizing interventions for multimorbidity involving common mental health conditions. In the study, the researchers aimed to determine the effectiveness of interventions included in RCTs for improving relevant mental health, physical health and quality of life outcomes. They further assessed risk of bias as part of their analyses.<br><br>The study's strengths include extensive analyses characterizing interventions, testing the effectiveness of interventions and assessing the quality of the studies included. The study is limited in that it was not able to analyze as part of the meta-analysis, physical health outcomes, due to heterogeneity in outcomes.<br><br>This article addresses a valuable question about the effectiveness of interventions for treating comorbid physical and mental health conditions, and has addressed it through various analyses, considering intervention timeframes, several different types of outcomes, different types of interventions and quality of evidence. It is notable that the researchers found little evidence of long-term effectiveness for most interventions.<br><br>MAJOR COMMENTS:<br><br>- The study would benefit from clarification about the screening and study selection approach used and further elaboration on several points in the methods, as indicated below in my specific comments.<br><br>SPECIFIC COMMENTS:<br><br>Abstract:<br>In the results of the abstract, the authors refer to organizational vs. patient-oriented interventions but did not make this distinction clearly in the methods.<br><br>Without the above distinction between organizational and patient-oriented interventions clear to the reader, the discussion in the abstract seems vague. Additionally, many of the findings showed no effects. The authors may want to consider modifying the discussion point in the abstract to reflect the mixed effectiveness found.<br><br>Introduction:<br>The introduction highlights the challenges and impact of multimorbidity that involves co-morbid mental health conditions; however, it does not provide any background on the gap that this systematic review aims to fill. The introduction would benefit from additional background on what is known about the effectiveness of interventions for multimorbidity with mental health conditions and what the gaps are that specifically pertain to the evidence this review is aiming to provide.<br>- For example, there is a systematic literature review and meta-analysis by Patel et al. 2024 (PMID: 37530738; DOI: 10.1093/fampra/cmad061) or another by Smith et al 2021 (https://doi.org/10.1002/14651858.CD006560.pub4)

	It would also be helpful if the authors provided some background on the types of interventions, i.e. care models and key components, that exist for treating multimorbidity with mental health conditions.<br><br>Methods:<br>Can the authors please add the link in a citation for the protocol on PROSPERO?<br><br>The screening and article selection process is not clearly described in the methods. Based on the PRISMA diagram, the researchers indicate that articles were removed by "automation tools" before screening, both to identify duplicates and those articles that are ineligible. Which automation tools were used and how did the tools determine eligibility?<br><br>Were there years of publication that the search was limited to? If so, can the authors please add this to the methods description?<br><br>Population, article type and study design reasons are indicated in the PRISMA diagram of search results, however, the inclusion criteria described in the methods have different and/or more criteria (e.g. intervention-related criteria) and are more nuanced (e.g. "multi-condition stipulation"). There seems to be a disconnect between the inclusion criteria and how articles were screened for eligibility. For greater transparency in the approach used for screening, can the authors please clarify the following?<br>- In the first phase of screening (I assume this is based only on title and abstract?), almost 94% of articles were excluded (5,973 out of 6,365), but there are no reasons provided for these exclusions. Can the authors please specify the reasons for exclusion in the first phase of screening?<br>- Which are the population-related inclusion criteria? Is it just that the population in any RCTs considered be focused on adults aged 18 years or older? Or does it also include the criteria about conditions? Additionally, can the authors please clarify how "symptoms of depression and/or anxiety" were considered when reviewing for eligibility, i.e. did patients included in clinical trials need to have mental health diagnoses as part of their study inclusion criteria?<br>- Please clarify the misalignment between the intervention-related criteria described in the methods and the fact that these were not indicated in the PRISMA diagram exclusion reasons. Also, were studies with interventions that were not part of the inclusion criteria excluded because of the "wrong study design"?<br>- The authors mention "outcomes of interest" under the inclusion criteria. Were the types of outcomes also considered for eligibility? If so, can the authors please elaborate on this criteria, and include it in the PRISMA diagram, if any studies were excluded based on it?<br><br>Did the two reviewers who extracted the data review independently, and if so, was agreement calculated between them? How was reliability in interpretation ensured?<br><br>When the researchers describe the categorization of interventions into organizational or patient-oriented (pp.6-7), can they please add the definitions for each category? Additionally, how were interventions categorized if they were multi-level interventions that involved both the organizational level and the patient level? It appears that these 2 categories were treated as mutually exclusive

	in the analyses, however, it is not clear how the categorization was defined and if there was any overlap.<br><br>In the description of the assessment timepoints in the methods, it would be helpful to specify the timelines used for each analysis (e.g. end of intervention = 4-12 months for organizational interventions).<br><br>Pertaining the methods description of RoB, there are a couple of clarifications that would be helpful:<br>- On p.6, line 52-53: Can the authors please specify the 5 domains from the RoB?<br>- Can the authors please elaborate on how they determined the overall RoB, beyond those studies that had high risk of any type of bias? For example, why would the overall risk of bias be the same for a study that has 'some concerns' in one of the domains and a study that has 'some concerns' for 4 of the 5 domains?<br><br>Related to the PPI group, how many members were included as part of this group? It would be helpful to specify this in the methods.<br><br>Results:<br>In the first sentence in the results, the authors state, "In total, 8663 potentially relevant titles were screened…"—were just titles of articles screened or titles and abstracts?<br><br>Table 1 – In table 1, there is a category for Depression and a separate category for Depression or anxiety under 'Mental health conditions included.' Are these mutually exclusive or is there some overlap? Can the authors please elaborate on how mental health conditions were determined and classified in the analyses? It also seems to be inconsistent with what is in Table 2, where depression and anxiety are separate<br><br>Table 2 – There are several letters indicating footnotes in the table ("a,b") but it seems like the letters are missing below in the footnotes to the table, or they are not clearly presented.<br><br>Discussion:<br>In the discussion on p.12, the authors note that one of the limitations of the meta-analysis is how the outcomes were treated in the analyses. I did not find the explanation of this in the methods to be particularly clear, and I think it would help if the authors added to the explanation of the analysis in the methods, elaborating on the approach they used for the outcomes, i.e. was a threshold used for all symptom scales?<br><br><br>MINOR EDITS:<br>p.7, line 52 – typo "focussed"<br>Sometimes the authors use passive voice and other times active voice in the writing. This can be checked and updated for consistency.

VERSION 1 – AUTHOR RESPONSE

Reviewer: 1

Comments:
Thank you for the opportunity to review this interesting and timely manuscript. My comments primarily focus on the methodology of the systematic review and the interpretation of findings from the exercise trials in patients with multimorbidity, which is my main area of expertise. Please find my detailed comments for the authors below.

Major Comments
R1.1 Inclusion Criteria: While the rationale for selecting depression and anxiety as representative mental disorders is clear, it would be helpful if the authors could further clarify the reasoning behind including any physical condition as part of the criteria. The intro states that there is a close association between mental health disorders and many (but not all) physical conditions.

We included studies targeting patients with depression or anxiety symptoms coexisting with any physical conditions (from a list of two or more in the trial eligibility criteria). This design was chosen in order to identify interventions orientated towards mental-physical multimorbidity, with potential for adaptability across different physical conditions. Limiting the scope of the review to interventions targeting specific physical comorbidities would also limit its applicability to a narrower group of patients, and therefore reduce its relevance to primary care commissioners and policymakers. While different physical conditions have varying levels of association with common mental disorders, there is evidence for an association with multimorbidity in general, including a dose-response relationship between number of long-term conditions and risk of depression. This justification for the inclusion criteria has been clarified in the introduction on pages 1 and methods on page 2.

R1.2 Rationale for Grouping: I commend the authors for providing detailed descriptions of the intervention components. While this is very useful for descriptive purposes, conducting a meta-analysis that combines all patient-oriented interventions and reporting a single SMD as primary results may be misleading. For example, although exercise, psychotherapy, and psychoeducation are all patient-directed interventions, they differ significantly in modality, underlying constructs, and rationale (as also noted by the authors in Box 2). This makes it challenging to analyse them together.

One of the aims of the study is to assess the effectiveness of existing interventions for multimorbidity involving depression or anxiety. Our analysis employs a broad followed by a narrow approach to intervention grouping to answer this question. While a single meta-analysis of all interventions was clearly not justifiable or meaningful, we feel that a synthesis across the two main groups of intervention is of value in providing a crude comparison between organisational and patient-oriented interventions, and is justified by the similarity of duration, intensity and complexity of interventions within the two groups. However, the differences between interventions within these two groups also complicates this synthesis, as we acknowledge in the limitations. This is reflected in the high level of heterogeneity in the meta-analyses, which underscores the need to undertake subgroup meta-analyses. However, the subgroup analysis itself is limited by the small number of studies within each subgroup, meaning meta-analysis was not always possible. We have reflected on this trade-off with additional text in the discussion on page 11 paragraph 2 and 3.

R1.3 Exercise Trials: Three trials are categorised as exercise trials (Figure 2 and Table S4). Two of these (Tsang 2006 and Tsang 2013) involve Qigong (traditional Chinese dance), while one (Lamberti) features a more “conventional” exercise program. Only the Tsang studies are included in the meta-analysis, so it may be inappropriate to draw conclusions about exercise in general, as these studies focus on Qigong. I would also like to draw the authors’ attention to a recently published trial on exercise for multimorbidity (MOBILIZE; https://www.nature.com/articles/s41591-025-03779-4), where I served as senior author. Please consider whether this study could enrich the introduction/discussion

on exercise for this population and provide a useful comparison, despite differences in the exercise interventions included in this review and the MOBILIZE trial.

The number of exercise intervention studies targeting mental-physical multimorbidity is notably limited, despite a strong evidence base in populations with multimorbidity and common mental disorders separately. This is a limitation that makes it difficult to draw conclusions about this subgroup, but we think this is an important finding given that it highlights a surprising evidence gap. This underscores why a broader initial synthesis across various patient-oriented interventions has value (see comment above).

Thank you for highlighting your paper which we have now cited in the discussion (page 12).

R1.4 Missing study from MA: The Lamberti study is not included in the meta-analysis. Upon reviewing the paper, it appears that the necessary data are available in Table 2 and the bar graph for SF-12. Since SF-12 is considered comparable to other HRQoL measures (Table S1), could the authors clarify whether it was not possible to estimate the SMD for this study?

This study reported effects on SF-12 mental component score (MCS) and physical component score (PCS) separately, but does not report an overall or composite SF-12 score. Neither the MCS or PCS are eligible for inclusion because these are sub-domains of quality of life and so not comparable with other measures of overall quality of life. Furthermore, it is not possible to simply add together the MCS and PCS scores, because the covariance, which is needed to calculate the standard deviation of the composite score, is not known.

R1.5 Also, is it feasible to estimate SMDs from other studies included in the Albatross plot (i.e., those excluded from the meta-analysis)? As SMDs can sometimes be estimated from p-values, graphs or other methods https://ma-accelerator.com/ should these studies be considered for inclusion in the meta-analyses?

We reviewed the studies that were not included in the meta-analysis with a statistician to see whether the reported results could in fact be used to estimate SMD, including using the web-tool that the reviewer has shared. In all cases the study authors had already been contacted to ask for provision of the optimal data for SMD (means and standard deviation for continuous outcomes). We followed Cochrane guidance by including both studies reporting raw results (mean and standard deviation) and those reporting adjusted effect size estimates, where the method and model had been clearly specified. However, we excluded studies where it was either not possible to estimate SMD based on the available results, or where estimation required assumptions we were not willing to make. The following four studies were excluded on this basis (and this detail has been added in Supplementary Box S3):

- Araya 2021 and Schrader 2007, which both reported results for depression as a dichotomous outcome (proportion achieving a specified reduction from baseline, or proportion with no vs mild vs moderate depression after treatment).
- Pumar 2019, which reported depression scores in the intervention and control groups as medians and interquartile ranges.
- Vera 2010, which reported results in the form of mixed-effects regression estimates, with insufficient information to confidently know how the model was specified.

R1.6 Risk of bias: I checked again the Lamberti study because in the Risk of Bias D5 domain it was deemed as Low risk of bias by the authors but I could not find their protocol in clinicaltrials.gov or similar database or in the original paper. Should this domain be deemed as 'some concerns' or 'high risk of bias' instead?

The risk of bias assessment has been reviewed. For this study (Lamberti 2022) the RoB-V2 question on whether study data had been analysed according to a pre-specified analysis plan had incorrectly been answered ‘probably yes’ instead of ‘no information’. This has now been corrected, downgrading this domain to ‘some concerns’. However, this did not affect the overall risk of bias result for this study, which was already ‘some concerns’. Other studies were checked for consistency, and two other similar errors found and corrected (Ell 2017, Johansson 2019). In both cases, these corrections also did not change the overall risk of bias result, only to the rating for domain 5. The corrected RoB diagram is shown on page 12 of the supplementary material.

R1.7 GRADE Assessment: I would like the authors to clarify the rationale for **NOT** downgrading for Inconsistency and Indirectness in most meta-analyses. For example, in patient-oriented interventions, the certainty of evidence is rated as moderate to high for depression. Given the limited number of trials, differences in interventions and the fact that inconsistency (heterogeneity) could not be explained by removing high-risk-of-bias studies or further investigation for example with meta-regression analyses, this rating may be overly optimistic. Also, is the population included in the RCTs representative of the broader target population (i.e., individuals with multimorbidity: depression/anxiety and at least one chronic condition)? If not, then one could consider downgrading also for Indirectness.

Considering these comments, we reviewed the GRADE assessments for each of the meta-analyses in the review, along with Cochrane guidance. We considered the characteristics of each study contributing to each synthesis. On reflection, we agree that the assessment of indirectness in the original submission was overly optimistic as it overlooked issues with a number of studies which affected their directness with respect to the research question (e.g., pertaining to participant age, physical conditions, setting, or comparator). As the reviewer highlights, this particularly affected the patient-oriented group of studies. For all six of the meta-analyses for this group (three outcomes, two time points) at least one-third of studies were affected by issues of directness. The certainty/quality of these meta-analyses has therefore been downgraded (Table 2 and Table S5) and the results (page 7/8) and discussion (page 9/10) have been updated accordingly.

R1.8 Change Scores Clarification: Could the authors please clarify whether the reported effects are based on end-of-treatment scores, change scores, or both?

We have clarified in the methods (page 4) that the reported effects are based on difference between the end-of-treatment scores for the intervention and control groups.

Minor Comments

R1.9 Consider briefly reporting the certainty of evidence in the Results section, rather than only in the Discussion (e.g., see the first paragraph of the Discussion).

The certainty/quality of evidence is now also reported in the results section (page 7/8).

R1.10 PPI: It is commendable that patients were involved in the systematic review. However, more detail on the number, characteristics, and representativeness of included participants would help readers understand and potentially reproduce this aspect of the methodology.

We have added detail on the number of individuals involved in the PPI activity (page 5), but, reflecting that PPI contributors are not research participants, and consistent with the Guidance for Reporting Involvement of Patients and the Public (GRIPP2-SF), we have not collected or reported individual characteristics, nor assessed representativeness.

R1.11 PRISMA Compliance: Please consider explicitly stating that the review follows the PRISMA

checklist.
This statement has been added on page 1.

R1.12 Comparator: Consider briefly describing the characteristics of the comparators in the Results section to better contextualise the intervention effects.

The results (page 6) now include a breakdown of the comparators across the 29 studies.

R1.13 Meta-Regression Analyses: Please mention that the heterogeneity observed in the meta-analysis could not be further explored, for example, via meta-regression analyses.

This clarification has been added in the methods on page 5.

Reviewer: 2

Comments:

This is a very interesting and carefully conducted study, focusing on a group of patients with physical–mental multimorbidity who represent some of the greatest complexity and risk of adverse outcomes in today's healthcare systems. The topic is highly relevant and timely, and I commend the authors for their rigorous work. Below, I provide several suggestions that may help strengthen the manuscript further:

R2.1 I recommend making it clear in the title that the focus is on primary care and/or community-based interventions, to better reflect the scope and narrow down the focus of the review.

We have updated the title to "Primary care and community interventions for multimorbidity involving depression or anxiety: A systematic review with meta-analysis".

R2.2 Basis for MPM targeting: The introduction notes that individuals with both physical and mental health conditions experience poorer quality of life and physical health outcomes, which justifies interventions targeting this group. However, it remains somewhat unclear how outcomes for people with both physical and mental health conditions differ from those with (i) only mental health conditions, or (ii) one mental and one physical condition. Strengthening the rationale for specifically targeting persons with combined physical–mental multimorbidity would make the case more compelling.

We agree that systematic review of interventions in other groups of people with multimorbidity would be useful, but this wasn't our purpose. We have provided a clearer justification for our choice in the first paragraph of the introduction:

> *Interventions targeting this high-need high-cost group are therefore a priority for patients and policy makers.[8]-[11] Moreover, while improving outcomes in people with multimorbidity in general is challenging,[12] a more targeted approach focussing on individuals with mental-physical multimorbidity has the potential to be more effective and cost-efficient,[13] while also addressing a problem which is highly socially patterned.*

R2.3 Choice of outcome: Measures of distress and mental components of well-being were not included, while several measures of physical well-being and self-reported health were considered. An

explanation for this asymmetry would be valuable. More broadly, the justification of outcome selection could be elaborated. Interventions of this type often include outcomes linked to psychosocial well-being, patient health behaviors, healthcare utilization, etc. While I understand the chosen end points, a stronger rationale for their prioritization would improve clarity.

The four outcome groups covered by this review – depression symptoms, anxiety symptoms, quality of life and physical health – were chosen to reflect important aspects of health and wellbeing for people with mental-physical multimorbidity, after discussion with the study PPI group. We sought a balance between including a broad range of measures that reflected these outcomes, while maintaining a precision and consistency of the underlying measurement constructs, in order to enable meaningful interpretation. In the case of the mental health outcomes, specific measures of depression and anxiety symptoms were chosen as these were commonly reported by the included trials (often as primary outcome) and had a specific, diagnostic focus since the same measures are often used to define or diagnose the presence of the common mental disorder. In contrast, other mental health measures were much more variably measured and reported.

There was far less consistency in the physical health outcomes being measured and reported, so it was not possible to take the same global (and meta-analytic) approach as for depression/anxiety. Although the certainty of the conclusions drawn from this part of the review is therefore lower, we believe this approach was the best way of achieving the objective (agreed with the PPI group) of assessing whether, in broad terms, interventions improved physical health outcomes in patients with heterogenous physical conditions. Health service use and economic impact, on the other hand, were beyond the scope of this study.

This rationale for the choice of measures has now been clarified in the methods, on page 3 (meta-analysis) and page 5 (combining p-values).

R2.4 Definitions table: I recommend adding a table summarizing definitions of each intervention type and subtype, to help readers navigate the evidence. I was surprised that self-management interventions were not highlighted among the patient-oriented approaches.

Definitions for each intervention type is given in Supplementary Box S2 and has been expanded in light of this comment (and could be incorporated in the main paper text at the editor's discretion). There were 17 interventions that included some element of self-management (based on the PRISMS taxonomy of self-management components), including four interventions categorised as psychoeducation for the purpose of this review, in which self-management was a dominant feature (this group was characterised by the delivery of education about depression/anxiety, the interplay with physical health, and the role of lifestyle and relaxation strategies, which could all be considered components of self-management for mental-physical multimorbidity). There were also aspects of self-management principles within five of the 12 psychotherapy interventions and in eight of the 10 organisational interventions.

The components matrix (Figure 2) shows the degree of overlap between intervention groups, and we have added a footnote to highlight the prominence of self-management within the psychoeducation group. The results (page 7, paragraph 3) also now emphasize this.

R2.5 Older populations: The exclusion of studies focused on older adults with mental health problems is concerning, given that this is often the group with the highest levels of complexity, due to multiple coexisting conditions, functional impairment, and unique manifestations of late-life mental health.

Could the authors provide the number of studies excluded on this basis, and at least a brief narrative summary of their findings?

It is an inherent limitation of systematic reviews that the inclusion criteria defined in the protocol create boundaries which lead to some studies being excluded despite potentially including evidence that is relevant to the research question. Five studies were identified during screening that involved primary care or community interventions targeting older people with depression, but which were excluded from the review because the presence of long-term physical conditions was not stipulated. This may have led to the exclusion of evidence that was potentially relevant to the research question. This limitation is highlighted in the discussion (page 11) and, in response to this comment, an overview of these five studies is now included in Supplementary Table S10.

R2.6 Discussion - Collaborative care: The role of collaborative care could also be discussed more explicitly, as it is often central in managing complex multimorbidity, and happens to be one of intervention types holding biggest potential.

The paper now includes an expanded discussion of collaborative care (pages 11-12) including the following:

> *Originally developed in the United States over 20 years ago as a model for managing depression in primary care,[101] collaborative care is an organisational intervention typically involving: [1] a multi-professional team (mental health professional, general practitioner and case manager); [2] enhanced interprofessional communication (regular meetings or shared records); and [3] a structured and patient-centred management plan involving evidence-based treatments (such as psychological therapy alongside physical health and medication reviews). Despite its strong evidence base, a number of barriers to real-world implementation of collaborative care have been identified, not least in terms of the complexity of organisational redesign it requires.[101,102]*

R2.7 Discussion – recommendations for intervention design: The authors conclude that the evidence for organizational and patient-oriented interventions is limited, particularly in the long term. It would be valuable if the discussion could be extended to recommendations for future intervention design, for example: content (should future approaches combine organizational and patient-oriented strategies?); duration (is longer follow-up, e.g., ≥2 years, needed?); target groups (should studies explicitly focus on multimorbid populations with ≥2 conditions, including at least one mental health condition?); and design (given that lack of blinding was a common source of bias, are there methodological strategies to address this?).

The section on implications for future research on page 12/13 has now been expanded, including the following:

> *Future research should explore possible how the implementation of effective yet complex interventions such as collaborative care can be supported within primary care systems…*
>
> *…On the other hand, psychotherapy interventions, which can be delivered in online and group formats, may offer a more feasible and inexpensive alternative to organisational interventions, while having similar short-term benefits. Future research should therefore explore how the early benefits of patient-orientated interventions might be sustained, including through the use of artificial-intelligence-driven conversational agents which have demonstrated potential in other clinical fields.[115]*

R2.8 Typos: Finally, I recommend a careful editorial review to correct small typographical errors that

appear throughout the text.
Thank you. The manuscript has now been thoroughly rechecked.

Reviewer: 3

Comments:
This SRMA aimed to identify effective interventions targeting individuals with multimorbidity, specifically those with depression/anxiety and comorbid physical conditions. The primary outcomes considered were improvements in quality of life and reductions in depressive and anxiety symptoms. The methodology is rigorous, with a well-conducted search strategy and clearly documented selection steps. The meta-analysis was performed to high standards, and the subgroup analyses are well justified.

R3.1 International framing: The introduction highlights the UK context, yet the SRMA itself is not geographically restricted. It is unclear why the UK is singled out in framing the background. Does the prevalence or healthcare organization in the UK differ in a way that justifies this focus? Similarly, the discussion refers specifically to NICE recommendations. Could these points be framed within a broader, more international context?

The introduction (page 1) and discussion (page 12) have both been expanded to address this point, including reference to global rather than UK estimates of prevalence for common mental disorders, and global approaches to care integration. The discussion of collaborative care (page 12) now also cites US, global and LMIC perspectives alongside UK guidelines.

R3.2 Link between the physical conditions and intervention effect: To enhance clinical relevance, the discussion would benefit from deeper consideration of which comorbid diseases—or clusters of diseases—interact most strongly with specific interventions. This would help clarify the mechanisms through which interventions exert their effects in multimorbid populations.

While a detailed meta-analysis by type of physical conditions was not possible with the available data, we have now expanded the discussion (page 9) as follows:

> *Almost half of the studies in this review (48%) specifically listed diabetes (plus at least one other condition) in their physical condition inclusion criteria, including all six of the studies of collaborative care. This pattern points to the particularly strong association between depression and diabetes that has been demonstrated in observational studies,[100] but may also reflect a degree of confirmation bias. More research is needed to examine whether interventions like collaborative care also improve outcomes in more heterogenous populations with multimorbidity.*

R3.3 Clustering: In the section on knowledge gaps and future directions, the concept of multimorbidity patterns or clusters is notably absent. Recent literature increasingly supports this approach as a way to stratify populations and identify the most appropriate target groups for interventions. Focusing only on individual comorbidities may not capture the complexity of multimorbidity, and patterns-based approaches could provide more meaningful insights for tailoring interventions.

This section (page 13) has now been expanded to include the following:

> *The high proportion of studies (particularly of collaborative care) which listed diabetes plus one other condition in their physical condition inclusion criteria highlights the need to consider both whether such interventions remain effective among a more heterogenous populations*

with multimorbidity, as well as whether certain clusters of mental-physical multimorbidity are more responsive to certain interventions.

Reviewer: 4

Comments:
I want to thank the authors for the opportunity to review their study on identifying and characterizing interventions for multimorbidity involving common mental health conditions. In the study, the researchers aimed to determine the effectiveness of interventions included in RCTs for improving relevant mental health, physical health and quality of life outcomes. They further assessed risk of bias as part of their analyses.

The study's strengths include extensive analyses characterizing interventions, testing the effectiveness of interventions and assessing the quality of the studies included. The study is limited in that it was not able to analyze as part of the meta-analysis, physical health outcomes, due to heterogeneity in outcomes.

This article addresses a valuable question about the effectiveness of interventions for treating comorbid physical and mental health conditions, and has addressed it through various analyses, considering intervention timeframes, several different types of outcomes, different types of interventions and quality of evidence. It is notable that the researchers found little evidence of long-term effectiveness for most interventions.

MAJOR COMMENTS:

R4.1 The study would benefit from clarification about the screening and study selection approach used and further elaboration on several points in the methods, as indicated below in my specific comments.

Additional detail on the screening and selection process has now been added to the paper, including a more detailed description of the reason for exclusion at full text stage as now shown in the PRISMA flow diagram (Figure 1). Specific clarifications and changes are detailed in response to the comments listed below.

SPECIFIC COMMENTS:

Abstract:
R4.2 In the results of the abstract, the authors refer to organizational vs. patient-oriented interventions but did not make this distinction clearly in the methods. Without the above distinction between organizational and patient-oriented interventions clear to the reader, the discussion in the abstract seems vague.

This categorisation is now mentioned within the methods section of the abstract.

R4.3 Additionally, many of the findings showed no effects. The authors may want to consider modifying the discussion point in the abstract to reflect the mixed effectiveness found.

The results now also include several of the no-effect results from the meta-analyses.

Introduction:
R4.4 The introduction highlights the challenges and impact of multimorbidity that involves co-morbid mental health conditions; however, it does not provide any background on the gap that this systematic

review aims to fill. The introduction would benefit from additional background on what is known about the effectiveness of interventions for multimorbidity with mental health conditions and what the gaps are that specifically pertain to the evidence this review is aiming to provide. For example, there is a systematic literature review and meta-analysis by Patel et al. 2024 or another by Smith et al 2021.

The introduction (page 1) has now been expanded to clarify the gap that this systematic review fills, which was previously only featured in the discussion. This includes the following passage in which the mentioned studies are cited:

> *Previous systematic reviews in this area have either focussed on specific comorbidity pairs,[23-25] specific interventions,[26,27] or on a range of interventions for multimorbidity, but without necessitating the presence of depression or anxiety.[12]*

R4.5 It would also be helpful if the authors provided some background on the types of interventions, i.e. care models and key components, that exist for treating multimorbidity with mental health conditions.

The introduction (page 1) has been expanded to highlight a number of interventions which have demonstrated potential for benefits to both physical and mental health, including those based on the collaborative care model, self-management principles, psychological therapies (particularly cognitive behavioural therapy) and physical activity. A more detailed description of collaborative care is also now included in the discussion on page 12.

Methods:

R4.6 Can the authors please add the PROSPERO link in a citation for the protocol?

The PROSPERO link on page 15 has now also been included as a citation when mentioned on page 2.

R4.7 The screening and article selection process is not clearly described in the methods. Based on the PRISMA diagram, the researchers indicate that articles were removed by “automation tools” before screening, both to identify duplicates and those articles that are ineligible. Which automation tools were used and how did the tools determine eligibility?

This refers to the use of the automated functions on Covidence which detect duplicates and non-RCT papers. These exclusions were checked for accuracy. This has been clarified within the footnotes of the PRISMA diagram.

R4.8 Were there years of publication that the search was limited to? If so, can the authors please add this to the methods description?

The database searches used unlimited lookback (i.e., from database inception) up to the date of the search (November 2024). This is stated on page 2.

R4.9 Population, article type and study design reasons are indicated in the PRISMA diagram of search results, however, the inclusion criteria described in the methods have different and/or more criteria (e.g. intervention-related criteria) and are more nuanced (e.g. “multi-condition stipulation”). There seems to be a disconnect between the inclusion criteria and how articles were screened for eligibility. For greater transparency in the approach used for screening, can the authors please clarify the following?

The reasons for exclusion at full text screening, shown in the PRISMA diagram (Figure 1), have now been updated to more accurately reflect the inclusion criteria and to give more nuanced breakdown.

R4.10 In the first phase of screening (I assume this is based only on title and abstract?), almost 94% of articles were excluded (5,973 out of 6,365), but there are no reasons provided for these exclusions. Can the authors please specify the reasons for exclusion in the first phase of screening?

These 5,973 studies were excluded at the first phase of screening based on clear and obvious information in the title or abstract that indicated ineligibility for the review. This included things like clearly being the wrong type of article (e.g., a protocol, conference abstract or review), clearly being the wrong population (e.g., trials in children, or in adults with mental disorder other than depression or anxiety), or clearly being the wrong intervention type (e.g., medication trials or in-patient interventions). It is typical for a large proportion of studies identified by a database search to be excluded at title and abstract screening in a systematic review, and it is consistent with PRISMA reporting guidelines not to give reasons for exclusion at this stage. However, in light of this comment, a footnote has been added to the PRISMA diagram giving additional information on this phase of screening.

R4.11 Which are the population-related inclusion criteria? Is it just that the population in any RCTs considered be focused on adults aged 18 years or older? Or does it also include the criteria about conditions?

The additional information now included in the PRISMA flow diagram (Figure 1) addresses this point, showing that the population-related criteria used for exclusion at full text screening included studies where the recruited population did not have depression or anxiety symptoms (or had other mental disorders beside depression/anxiety); where the presence of a long-term physical condition was not stipulated; or where the study only recruited individuals with a specific mental-physical comorbidity pair (e.g., cancer with anxiety).

R4.12 Additionally, can the authors please clarify how “symptoms of depression and/or anxiety” were considered when reviewing for eligibility, i.e. did patients included in clinical trials need to have mental health diagnoses as part of their study inclusion criteria?

For inclusion in the review, studies needed to *target* patients who were identified as having symptoms of depression or anxiety. The methods section on page 2 has been expanded to clarify that this included studies where all recruited participants had a formal diagnosis of depression or anxiety, as well as others where participants’ symptoms of depression or anxiety were above a given threshold on a validated screening tool. Table 2 gives the specific mental health criteria for each study (e.g., PHQ9≥10).

R4.13 Please clarify the misalignment between the intervention-related criteria described in the methods and the fact that these were not indicated in the PRISMA diagram exclusion reasons. Also, were studies with interventions that were not part of the inclusion criteria excluded because of the “wrong study design”?

The additional information now included in the PRISMA flow diagram (Figure 1) addresses this point. This review intentionally took a broad, inclusive approach to interventions, so the only intervention-related exclusion criteria (page 2) were medication-only trials, and hospital inpatient interventions.

Most studies that failed these criteria were easily identifiable at title or abstract screening, with only two studies excluded at full text stage due to ‘wrong setting’.

R4.14 The authors mention “outcomes of interest” under the inclusion criteria. Were the types of outcomes also considered for eligibility? If so, can the authors please elaborate on this criteria, and include it in the PRISMA diagram, if any studies were excluded based on it?

Studies had to report mental health, physical health or quality of life outcomes to be eligible for inclusion. Studies reporting only economic analyses were excluded. This has been clarified at the bottom of page 2. The specific outcome measures included in the three domains of interest are detailed on pages 3 (mental health, quality of life) and page 5 (physical health). The PRISMA flow diagram (Figure 1) also now shows that 18 studies were excluded at full text screening due to ‘wrong outcomes’, referring typically to studies where only economic analyses were reported.

R4.15 Did the two reviewers who extracted the data review independently, and if so, was agreement calculated between them? How was reliability in interpretation ensured?

This process has now been clarified in the methods on page 3 as follows:

> *Initial data extraction was conducted by two reviewers (KS plus a second) in order to refine the extraction form (i.e., agreeing on variable columns and consistent terminology) and ensure consistency in the level of detail and terminology. Two thirds of subsequent data extraction undertaken by KS was then checked by a second reviewer, who independently reviewed the study results to identify any discrepancies to be resolved by consensus.*

R4.16 When the researchers describe the categorization of interventions into organizational or patient -oriented (p.6-7), can they please add the definitions for each category? Additionally, how were interventions categorized if they were multi-level interventions that involved both the organizational level and the patient level? It appears that these 2 categories were treated as mutually exclusive in the analyses, however, it is not clear how the categorization was defined and if there was any overlap.

The definitions for each intervention category have been clarified in Supplementary Box S2, which could be included in the main text at the editor’s discretion. The section on intervention components (page 7) also now clarifies the key differences and degree of overlap between the organisational and patient-oriented intervention, which is also illustrated in Figure 2.

R4.17 In the description of the assessment timepoints in the methods, it would be helpful to specify the timelines used for each analysis (e.g. end of intervention = 4-12 months for organizational interventions).

This detail has now been added to the methods on page 4, as well as in the footnote for Figure S3.

R4.18 Pertaining the methods description of RoB, there are a couple of clarifications that would be helpful:
- On p.6, line 52-53: Can the authors please specify the 5 domains from the RoB?

This detail has been added on page 5.

R4.19 Can the authors please elaborate on how they determined the overall RoB, beyond those studies that had high risk of any type of bias? For example, why would the overall risk of bias be the same for a study that has ‘some concerns’ in one of the domains and a study that has ‘some concerns’ for 4 of the 5 domains?

Overall risk of bias was assessed using the template and algorithm of the Cochrane RoB-V2 tool, where the overall risk of bias is determined by the worst score in any of the five domains. We have expanded the description of the RoB assessment to add clarity on this point without wishing to exceed a reasonable word limit.

R4.20 Related to the PPI group, how many members were included as part of this group? It would be helpful to specify this in the methods.

This detail has been added on page 5.

Results:
R4.21 In the first sentence in the results, the authors state, "In total, 8663 potentially relevant titles were screened…"—were just titles of articles screened or titles and abstracts?

This has been corrected to now state titles and abstracts.

R4.22 Table 1 – In table 1, there is a category for Depression and a separate category for Depression or anxiety under 'Mental health conditions included.' Are these mutually exclusive or is there some overlap? Can the authors please elaborate on how mental health conditions were determined and classified in the analyses? It also seems to be inconsistent with what is in Table 2, where depression and anxiety are separate.

Table 1 describes the characteristics on the 29 studies, including their mental health inclusion criteria. In some cases, trials included participants with either depression or anxiety (alongside a long-term condition). In other cases, trials only recruited participants with symptoms depression (so patients with symptoms of anxiety but not depression were not eligible). Table 2 is a summary of the meta-analyses, so in this context, depression and anxiety refer to the trial outcomes, and these are separate because the effects of the interventions on anxiety symptoms (measured using defined, validated outcomes measures for anxiety) were meta-analysed separately from the effect on depression symptoms (measured using defined, validated outcome measures for depression). A footnote has been added to Table 1 to clarify this distinction. In addition, clarification has been added in the methods (page 2) regarding the review's inclusion criteria with respect to the presence of depression and anxiety within trial populations.

R4.23 Table 2 – There are several letters indicating footnotes in the table ("a,b") but it seems like the letters are missing below in the footnotes to the table, or they are not clearly presented.

Apologies, the footnotes used superscript for these letters which wasn't easy to read, and we have now enlarged them.

Discussion:
R4.24 In the discussion on p.12, the authors note that one of the limitations of the meta-analysis is how the outcomes were treated in the analyses. I did not find the explanation of this in the methods to be particularly clear, and I think it would help if the authors added to the explanation of the analysis in the methods, elaborating on the approach they used for the outcomes, i.e. was a threshold used for all symptom scales?

Clarification on this point has now been added in the methods on page 4 as follows:

> *For each study and each outcome, an estimate of the effect of the intervention was calculated based on the difference between the mean outcome scores of the intervention and control groups at the relevant assessment point. This effect was estimated as a standardised mean difference (SMD, the mean difference divided by the pooled standard deviation) to account for the fact that different outcome measures for the same construct (e.g., PHQ-9 and GDS for*

depression) have different scales. Where studies reported SMD with associated variance, this was extracted directly (adjusted if reported; unadjusted if not) as recommended by Cochrane guidance.[48] A pooled estimate of effect for the studies in each meta-analysis was calculated using a random effects model to account for heterogeneity among studies. Studies only reporting effect using dichotomous outcomes (such as the proportion of participants achieving a specified level of symptom improvement) were not included in the meta-analysis.

MINOR EDITS:
R4.25 p.7, line 52 – typo “focussed"

I believe ‘focussed’ is British English spelling, which I have tried to use consistently, but I am happy to change to the journal style if ‘focused’ is preferred.

R4.26 Sometimes the authors use passive voice and other times active voice in the writing. This can be checked and updated for consistency.
This has been corrected.

VERSION 2 – REVIEW

**REVIEWER NAME**	Kirkham, Jamie
**REVIEWER AFFILIATION**	The University of Manchester, Biostatistics
**REVIEWER CONFLICT OF INTEREST**	None
**DATE REVIEW RETURNED**	13-Jan-2026

**GENERAL COMMENTS**	I reviewed this paper as part of the BMJ manuscript meetings as a stats editor. Methodologically and statistically, this is a well-conducted systematic review which conforms to all good research practices. I have no further comments. I did note that the search strategy was over a year out of date but I will leave this as an editorial decision given this paper has been considered across the portfolio of journals for some time.<br><br>As part of the review, I also reviewed the responses to peer review comments which I felt were adequately addressed. I am supporting of this version.
